# Spatial-temporal dynamics of hunter effort for wild turkeys in Michigan

**DOI:** 10.1371/journal.pone.0230747

**Published:** 2020-04-01

**Authors:** Bryan S. Stevens, David R. Luukkonen, C. Alan Stewart, William F. Porter, James R. Bence, Michael L. Jones

**Affiliations:** 1 Department of Fisheries and Wildlife, Michigan State University, East Lansing, Michigan, United States of America; 2 Michigan Department of Natural Resources, Lansing, Michigan, United States of America; Institute for Advanced Sustainability Studies, GERMANY

## Abstract

Wild turkeys (*Meleagris gallopavo*; hereafter turkeys) are an important game animal whose popularity among hunters has increased in recent decades. Yet, the number of hunters pursuing turkeys appears to be in flux, patterns of hunter abundance have primarily been described at broad spatial scales, and the ability of management to impact hunter numbers in the post-restoration era of management through opportunity for quality hunting is unclear. We used county-scale estimates of turkey hunter numbers collected over a 14-year period (2001–2014) and time-series analyses to evaluate the spatial scales at which spring and fall turkey hunter populations fluctuate, and also used generalized linear mixed models to evaluate whether attributes related to quality turkey hunting explain recent patterns in hunter abundance. We found heterogeneity in turkey hunter population growth at finer spatial scales than has been previously described (i.e., counties and management units), and provide evidence for spatial structuring of hunter population dynamics among counties that did not always correspond with existing management units. Specifically, the directionality of hunter population change displays spatial structure along an east-west gradient in southern Michigan. We also found little evidence that factors providing opportunity for quality turkey hunting had meaningful impacts on recent spatial-temporal patterns of hunter numbers. Our results imply that providing quality turkey hunting opportunities alone may be insufficient for sustaining populations of turkey hunters in the future, and that modern determinants of hunter participation extend beyond the availability of abundant turkey populations. Moreover, our results demonstrate that interpretation of harvest data as indices of abundance for turkey populations is difficult in the absence of hunter effort data, as changes to turkey harvest are a function of potentially fine-scaled changes in populations of hunters, not simply changes to turkey populations.

## Introduction

Wild turkeys (*Meleagris gallopavo*; hereafter turkeys) are a prolific game animal for which hunting popularity has increased in recent decades. Turkey hunter numbers in the United States (U.S.) increased throughout the 1970s-1990s in response to rapidly growing turkey populations and the liberalization of hunting opportunities that followed [1,2,3,4]. Thus, in the face of general declines of hunter participation and concerns over wildlife management funding models that ensued [4,5,6,7,8], the number of hunters pursuing turkeys actually increased. For example, participation in spring turkey hunting in North America increased by 21% from 1994–1999 and by 6% from 1999–2004 to approximately 2.8 million hunters [3,9]. Changes to turkey hunter participation were not uniform in space, however, and parts of the Midwestern U.S. saw increases to spring turkey hunter participation of > 30% from 1996–2006 [4].

The number of spring and fall turkey hunters in the U.S. appeared to plateau after the successful completion of broad-scale restoration efforts for turkeys (~ year 2000) [2], but patterns of hunting participation remained highly variable [3,10,11]. Spring hunter participation decreased by 2% within the U.S. from 2009 to 2014, with state-level changes in hunter numbers as extreme as 57% losses (NV) and 77% gains (MN) over the same period [10]. Average turkey hunter effort (hunter days/year) for both spring and fall seasons decreased across the Midwestern U.S. in the post-restoration time period, yet spatial variation in effort patterns was prevalent, with some states (e.g., MO) showing increased effort for one or both seasons [11]. At a national level, fall turkey hunter participation increased by 10% within the U.S. from 2008 to 2013, with state-level changes in hunter numbers as extreme as 67% losses (NJ) and 159% gains (NM) over the same period [10]. Moreover, some states have shown differing trends in hunter effort between spring and fall hunting seasons in recent years (e.g., declining spring but increasing fall effort in MI) [11].

Recent studies provided important context for the management of turkey harvests by describing changes to hunter numbers over broad scales, yet most of this work has not provided a fine-grained depiction of changes at regional and local scales (but see [12]). Although hunting participation may be declining collectively across the U.S., the mechanisms affecting fluctuations of hunter numbers and the spatial scales at which hunter numbers fluctuate are not clear, and therefore the appropriate scale for monitoring hunter numbers is unknown. Broad-scale descriptions may not accurately characterize changes to turkey hunter populations at finer scales within a state or management region of interest. Drivers of change in turkey hunter populations are likely complicated and are poorly understood. Human dimensions surveys have documented attributes that commonly result in satisfied turkey hunters (e.g., low hunter interference, high hunter success, interaction with gobbling males) [13,14,15,16,17]. Yet it remains unclear whether these attributes have had meaningful effects on hunter numbers in recent years, or if turkey hunter populations are effectively fluctuating independently of hunting quality. Many factors, some under management control (e.g., regulation structures) but many not (e.g., human population density, amount of public land), may be contributing to recent fluctuations in turkey hunter numbers.

Understanding the scales of hunter population change and the ability of managers to affect change in these populations has strong implications for turkey monitoring and management programs. Monitoring programs in many states use harvest-based metrics to index changes in turkey populations over space and time. Using harvest-per-unit effort as a population monitoring index requires assumptions about the relationships between harvest, hunter effort, hunter effectiveness, and turkey population size, that generally are not tested [11,18]. Many states do not collect fine-scale data on hunter effort, and some do not collect any data on hunter effort and therefore rely on harvest alone for monitoring turkey populations. Use of unadjusted harvest to index turkey abundance relies on even more assumptions than harvest-per-unit-effort, including constant (or at least spatially and temporally unstructured) hunter effort within a region and timeframe of interest. Understanding the spatial scales at which hunter populations change is therefore vital to understanding the reliability of existing monitoring programs for turkeys. Moreover, understanding how management, beyond simply the setting of an upper bound (e.g., via quota systems), effects change in hunter populations is critical to recruitment and retention efforts. If factors that create good turkey hunting also explain spatial-temporal changes to hunter populations, then creating the opportunity for quality turkey hunting would be expected to result in increased hunter participation. Therefore, our objective was to answer 2 questions relative to the spatial-temporal dynamics of turkey hunter populations: 1) At what spatial scale do turkey hunter populations fluctuate? and 2) Do factors known to provide opportunity for successful and high quality turkey hunting explain recent changes in hunter populations? We hypothesized that the spatial scales of hunter population change would not be the same as the broad scales at which hunter numbers are typically monitored and predicted a finer-grained structure to changes in hunter populations. We also hypothesized that fine-scale changes in hunter numbers would reflect changes in hunting quality, and therefore predicted that changes in hunter participation would reflect changes in metrics that are commonly used to monitor turkey hunting quality (e.g., hunter interference and success rates).

## Materials and methods

### Ethics statement

No approval for use of human subjects was needed for this project because no human subjects were directly involved. The analyses (described below) did not rely on data from individuals, but rather on summary estimates of the total number of people that participated in hunting for a given location and year. Thus, no hunter-level information was collected or used in this project.

### Study area

**Fig 1:**
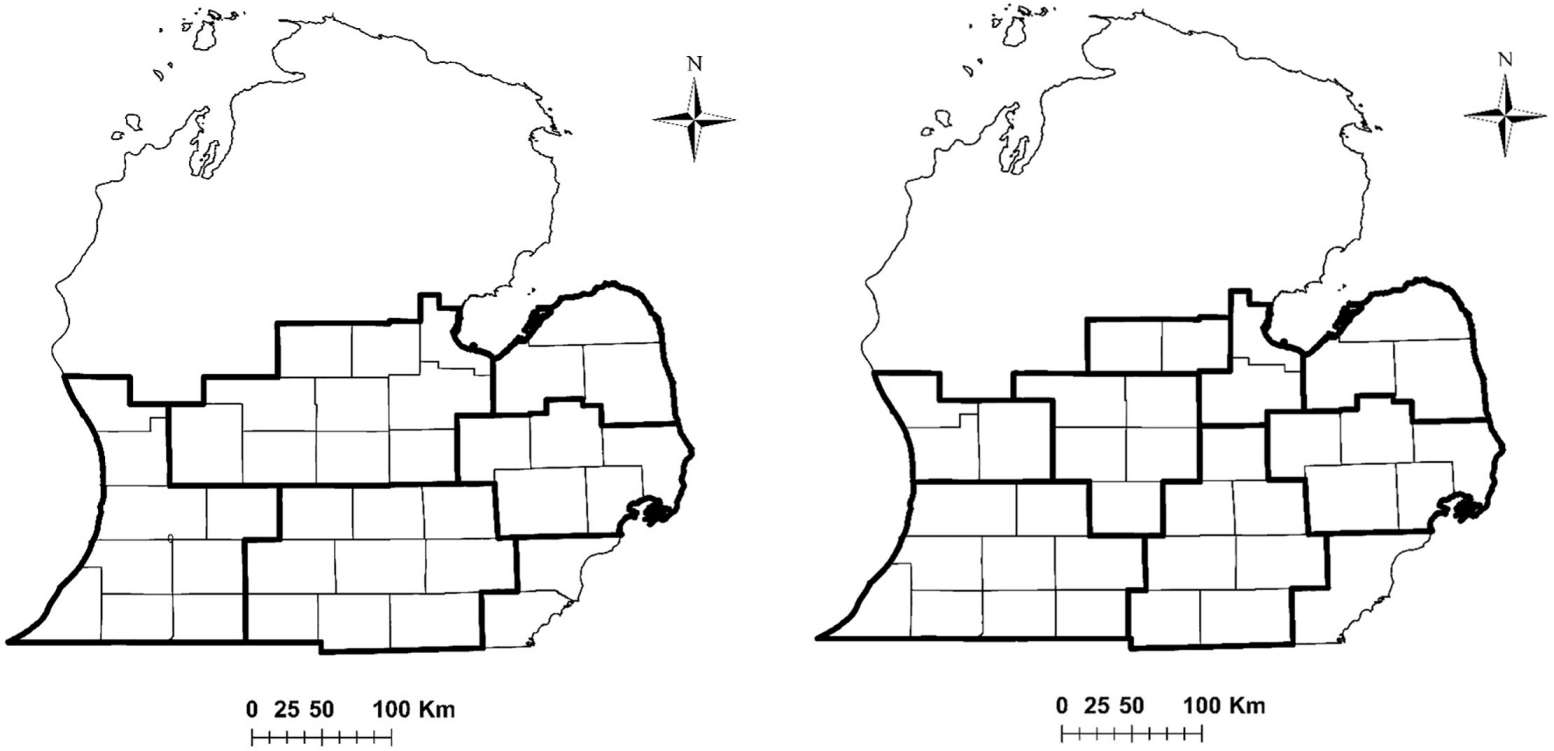
Wild turkey harvest management regions and counties open to spring (A) and fall (B) hunting in southern Michigan, USA, where we assessed dynamics and drivers of wild turkey hunter populations. Management regions are outlined in bold to denote their location within the lower peninsula of Michigan, and counties open to hunting are indicated (thin black outline).

We studied the spatial-temporal dynamics of spring and fall turkey hunters within the region approximating the ancestral range of turkeys in southern Michigan (Fig 1), which also includes the majority of turkey hunting activity within the state [19]. This area includes the 38 counties of southern Michigan open to spring hunting throughout the study duration (2001–2014) and the 36 counties that were also open to fall hunting (Detroit area excluded). This region encapsulated a range of human population densities, from dense urban areas to less populated rural and agricultural landscapes. Vegetation consisted primarily of a mixed agricultural-forest mosaic, with scattered wetlands and grasslands interspersed. The region is dominated by private land ownership, with smaller amounts of public lands open to hunting interspersed throughout.

### Data

#### Hunter population data

We used data collected annually by the turkey harvest monitoring program of Michigan Department of Natural Resources (MDNR) to assess spatial-temporal changes in spring (2001–2014) and fall (2002–2013) turkey hunter populations. Post-season mail surveys were sent annually to randomly selected license buyers for both spring and fall seasons ($\bar{x}$ = 12,680 and $\bar{x}$ = 4,764 for spring and fall respondents, *range* = 7,325–19,635 for spring and *range* = 3,736–7,719 for fall). These data were used to generate estimates of county-scale hunter population abundance (i.e., the number of hunters participating in a given county) for each season [20,21]. The proportions of annual respondents were generally high ($\bar{x}$ = 0.70 and $\bar{x}$ = 0.77 for spring and fall, *range* = 0.61–0.83 for spring and *range* = 0.57–0.91 for fall), and thus non-response bias was not likely to be substantial for estimates of hunter populations. We used annual, county-scale estimates of hunter populations produced by MDNR as the finest resolution for our analyses (S1 File), and thus no hunter-level information was used, nor did we assess behavior or magnitude of effort within a hunting season (e.g., days hunted) for individual hunters. Estimates of county-scale turkey hunters (those that participated in turkey hunting) were used instead of license sales numbers, which lack the same level of information provided by the county-scale estimates. License sales do not ensure participation, whereas survey data provide direct estimates of those license purchasers who participated in hunting. In addition, license sales data lack the spatial context provided by hunter population estimates, which estimate hunter participation at a county scale.

#### Covariate data

We hypothesized multiple factors that provide the opportunity for successful and high-quality turkey hunting would affect spatial-temporal patterns of hunter populations (Table 1; S1 File). We hypothesized that increased hunting opportunity in the form of increased season length (no. days) and increased area of land accessible for public hunting would positively affect hunter numbers (Table 1). We also hypothesized previous perceptions of hunting quality would affect dynamics of hunter populations (Table 1). Hunter success, interference, and satisfaction were all ascertained via questions included in mail surveys and annually estimated at the county scale from spring survey data (i.e., fraction of successful hunters, fraction of hunters reporting interference, and fraction of hunters reporting that they were satisfied) [20,21]. Hunter satisfaction data were not collected from fall harvest surveys and data on interference during fall were not collected regularly, hence neither of these factors were used as explanatory variables for analyses of fall hunter population size. We hypothesized the fraction of successful turkey hunters during a spring or fall hunting season would positively affect participation in the following spring or fall season respectively. We also hypothesized across season effects of hunter success, where success during fall and spring would affect participation in the next open season. We hypothesized the fraction of turkey hunters reporting interference by other hunters during spring turkey hunting would negatively affect participation in the following fall and spring seasons. Lastly, we hypothesized the fraction of hunters reporting they were satisfied in a given spring season would positively affect participation in the fall season of the same year, as well as the following spring season.

**Table 1:** Covariates used, and hypothesized direction of influence of each covariate on county-scale wild turkey hunter numbers in southern Michigan, USA, during spring and fall hunting seasons.

Covariates by category	Direction of influence
Hunting regulations^a^	
Season length	+
Hunter perceptions	
Hunter success^b^	+
Hunter interference^c^	-
Hunter satisfaction^d^	+
Access to public lands	
Area of public hunting land in county	+
Abundance of people	
Human population size^e^	-
Turkey population dynamics^f^	
Density of male turkeys	+
$\hat{\lambda }$	+

^a^ Other commonly used regulatory tools were mostly invariant through space and over the study period. For instance, bag limits were invariant for spring hunting. Also, the quota of turkey licenses was never achieved, and as such the availability of licenses was effectively unlimited to potential turkey hunters.

^b^ Fraction of turkey hunters in county that harvested a turkey the previous spring or fall, respectively. Covariates for county-level success across seasons were also considered, where success the previous fall (spring hunter models) and spring (fall hunter models) were included in hypothesized models.

^c^ Fraction of turkey hunters in county reporting interference by other hunters during turkey hunting activities the previous year. This information is only available for spring hunting seasons.

^d^ Fraction of turkey hunters in county reporting quality of hunting experience as excellent, very good, or good (from categorical response that also included fair and poor as options). This information is only available for spring hunting seasons.

^e^ Estimated population of people residing in county by year.

^f^ Density of male turkeys (N^Area) at the start of spring hunting and the finite rate of change in male abundance at the start of spring ($\hat{\lambda }$) were estimated at the management-unit scale. Turkey density at the start of the previous spring (*t-1*) and the finite rate of change from 2 springs prior to the previous spring (*t-2 → t-1*) were hypothesized to influence hunter effort during spring of the current year *t*. In contrast, spring turkey density during the current year, and turkey population finite rate of change from the previous spring to the current year’s spring (*t-1 → t*) were hypothesized to influence hunter effort during the fall of the current year.

In addition to public access and hunter perceptions of hunting quality, we hypothesized that populations of humans and turkeys would affect spatial-temporal patterns of turkey hunter numbers (Table 1). We hypothesized that increasing human population within a county would negatively affect county-level hunter participation in both spring and fall seasons, as increased human populations are likely to be associated with urbanization and other land uses that exclude hunting. However, we acknowledge that this prediction is scale specific; whereas more people residing in a county may result in reduced county-level hunter participation, a larger pool of inhabitants at broader scales (e.g., state or management region) may result in more hunters as measured at those scales. We obtained county- and year-specific estimates of human abundance from U.S. Census Bureau data that are freely available online (population and housing unit estimates, https://www.census.gov/programs-surveys/popest/data/tables.html, accessed 2/2016).

We also hypothesized that higher turkey density, as well as increasing turkey populations in the short term would positively affect county-scale hunter populations, as both of these metrics are likely to result in increased numbers of interactions with turkeys when hunters go afield (and therefore greater abundance of turkeys perceived by hunters). We used existing estimates of annual male turkey abundance at the start of spring hunting at the management unit scale (Fig 1A) and included estimates of male turkey abundance and population growth as covariates. Specifically, we hypothesized that male turkey density at the start of the previous spring (*t-1*) and population growth from 2 springs prior to the previous spring (*t-2 → t-1*) would positively affect hunter numbers during spring of the current year *t*. We also hypothesized that spring turkey density during the current year, and population growth from the previous spring to the current year’s spring (*t-1 → t*) would positively affect hunter numbers during the fall season of the current year *t*.

### Statistical analyses

#### Overview

We conducted two distinct types of analyses using county-scale hunter population estimates. First, we explicitly modeled hunter population change using state-space models. These analyses evaluated whether hunters should be modeled as a single population changing over the study region, as one unique population for each management unit, or as distinct populations for each county, and thus were intended to illuminate the spatial scales of hunter population change. The second analyses used generalized linear mixed effect regression to determine which covariates might explain temporal or spatial heterogeneity in county-scale hunter numbers, and thus were intended to evaluate hypothesized drivers of hunter population change.

#### Modeling hunter population change

We used multivariate auto-regressive state-space methods (hereafter MARSS models) [22] to model spatial-temporal dynamics of spring and fall turkey hunter populations, and to identify the spatial scales at which hunter populations fluctuate. Models of identical form were fit separately to data for spring and fall hunters. MARSS models are structural time series models that treated population-scale hunter numbers as non-stationary stochastic populations using multivariate dynamic linear models. Using MARSS models, we accounted for both dependence of hunter numbers over time within a population and spatial dependence of annual hunter number fluctuations among populations. Herein we sketch out the main aspects of our models, with additional mathematical detail presented in S1 File.

We considered populations as specific to each county, specific for each multi-county management-unit, or as a single overall population describing dynamics for the study region (i.e., all 38 counties in southern Michigan were a single population). Our population models used a stochastic exponential model to describe growth of hunter numbers (subscript for population suppressed for simplicity):

$$n_{t}=n_{t-1}e^{u+w_{t}},$$ (1)

for *n* hunters in year *t*, with long-term population growth rate *u* and stochastic fluctuation in that growth rate *w*_*t*_. We rewrite this on the log scale:

$$x_{t}=x_{t-1}+u+w_{t},$$ (2)

where *x*_*t*_ and *x*_*t*−1_ represent the natural log of hunter abundance at times *t* and *t-1*, respectively.

When modeling multiple populations (county-level or management-unit level) the process errors (*w*_*t*_) were allowed to be correlated among populations. We considered four alternative plausible approximations for the process-error covariance structures: 1) unconstrained, with unique process-error variances for each population and unique correlations for the process errors for each pair of populations; 2) equal variance-covariance among populations, with the same process error variance for all populations and the same correlation for all pairs of populations; 3) diagonal and unequal, where variances varied among populations and correlations were zero; and 4) diagonal and equal, where all populations shared the same process-error variance and correlations were zero.

When populations were modeled for each county, the observation model simply added an observation error to the observed state (county subscripts suppressed):

$$y_{t}=x_{t}+v_{t}$$ (3)

When there was not direct correspondence between the county-level observation (i.e., estimate of hunter abundance) and the population being modeled (e.g., management region or study area) we needed to keep track of which population contributed to the observation in a specific county:

$$y_{Ct}=x_{Pt}+a_{C}+v_{Ct}$$ (4)

Here *y* is the log-scale estimated hunter numbers in a county and year, *C* denotes county, *P* denotes population, and *v* is the county and year specific observation error. The nuisance parameter, *a* is needed to calibrate the observation to the population scale because each population is contributing to observations in multiple counties (and the fraction of that population contained within each county varies among counties; see [22]). Given that survey data were collected independently for different counties we assumed observation errors were independent among counties. We considered two alternative models for the observation error variances: 1) one shared variance value that was equal for all counties, and 2) unique, county-specific variances.

For both the spring and fall seasons we considered 18 alternative models: 1) 8 models with county-scale population dynamics (4 process-error models × 2 observation-error models), 2) 8 models with management-unit scale population dynamics (4 process-error models × 2 observation-error models), and 3) 2 models with study-area scale dynamics (2 observation-error models). We compared support for hypothesized models separately for spring and fall seasons using Akaike’s Information Criteria corrected for small sample sizes (AIC_c_) [23].

We fit all models via the MARSS package [22] using maximum-likelihood estimation in program R version 3.2.2. We fit all models using the default Expectation-Maximization (EM) algorithm used by the MARSS package [24], with 10 random sets of starting parameter values for each model to ensure convergence to global maxima of the likelihood surface. We also attempted model fitting via the BFGS algorithm if convergence was not achieved with the EM algorithm [22]. We considered a model unsupported if convergence was not achieved with either computational method.

#### Explaining patterns of hunter population size

We used generalized linear mixed effects regression to evaluate support for covariates hypothesized to affect spatial-temporal patterns of county-scale spring and fall hunter populations. We analyzed covariate effects using the negative binomial model, as opposed to using log-transformed hunter counts and the normal distribution, to guard against the potential impacts of data overdispersion on inferences about covariate effects [25]. We conducted analyses separately for spring and fall seasons, using 3 stages for each analysis. First, we identified the appropriate distributional and random effects structure. Second, we reduced the number of hunter perception and turkey population dynamics covariates (and therefore final candidate models) by comparing support for univariate models. Third, we generated a final candidate model set and evaluated support for these models using model selection. We also included quadratic time trends in all regression models to account for potentially non-linear changes in average hunter abundance over the study duration, as suggested by study-area wide patterns of hunter effort (S1 Fig).

In the first stage of analysis we considered 8 alternative models for random effects and distributional assumptions, and fit these alternatives using the global model for fixed effects (i.e., the model that included all fixed effects). Models were ranked using AICc, and the top random effects and distributional structure for each data set of estimated county-scale hunter abundance was used in all further analyses. We evaluated 4 plausible spatial-temporal random effects structures: 1) random intercepts by county, 2) random intercepts by year, 3) random intercepts and time trend slopes by county, 4) random intercepts by county and year with random time trend slopes by county. In addition, the negative binomial is a flexible family of statistical distributions with multiple unique parameterizations, including models with linear and quadratic variance-mean relationships [26,27]. Thus, we fit each of the 4 random effect models using both the linear and quadratic variance-mean parameterizations, yielding the 8 alternatives.

Because of the large number of hypothesized covariates (Table 1), in the second stage we conducted univariate analyses to reduce the number of variables describing hunter perceptions and turkey population dynamics. We compared univariate models for hunter perception covariates (hunter success during previous spring and fall seasons, hunter interference during the previous spring season, and hunter satisfaction during the previous spring season) using AICc and retained covariates in the final model set if they were ≤2 ΔAICc units of the top model. Similarly, we compared univariate models for turkey population dynamics covariates (population density and growth rate) using AICc and retained covariates with ΔAICc ≤ 2 for the final model set. This resulted in 2 hunter perception covariates and 1 turkey population covariate retained for spring analyses, and 1 hunter perception covariate and 2 turkey population covariates retained for fall analyses (S2 Table & S3 Table). Lastly, in the third stage these variables were combined with the other hypothesized covariates (Table 1) to generate a final model set of 62 and 32 candidate models to explain patterns of spring and fall hunter effort, respectively. We fit all mixed models using AD Model Builder (ADMB) [28] called from within R version 3.2.2 using the glmmADMB package [29].

## Results

**Fig 2:**
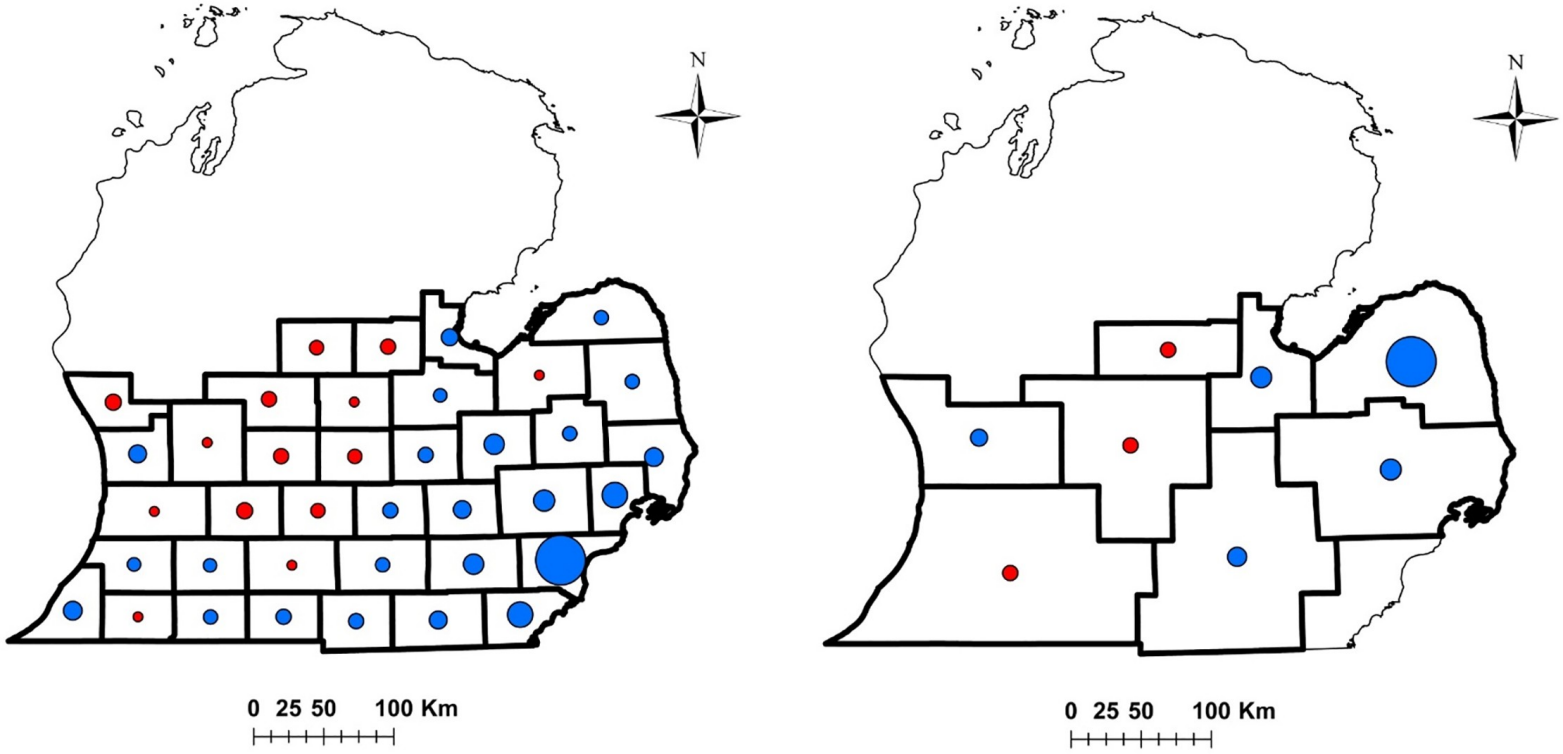
Change in wild turkey hunter populations for spring (A) and fall (B) hunting seasons in southern Michigan, USA. Colors indicate positive (blue) or negative (red) changes in hunter populations, whereas the sizes of circles within counties are proportional to the rate of hunter population change.

Our MARSS analyses suggested the scale of dynamics for turkey hunter populations operated at a county and management unit within southern Michigan (Table 2, Fig 2). The top MARSS model for spring hunter populations contained county-scale growth, whereas the top fall model included management-unit-scale growth, and there was little model-selection uncertainty for either season (*w*_*i*_ = 1.0 and 0.94 for the top spring and fall models, ΔAIC_c_ ≥ 299 and ≥ 5 between top and remaining spring and fall models; Table 2). Top models included the equal process-error variance-covariance matrix (Table 2), with a shared variance and single pairwise covariance parameter describing correlations of population fluctuations among counties (spring) or management units (fall), indicating temporal fluctuations in hunter numbers were of similar magnitude and were correlated to a similar degree over time among counties (spring) and management units (fall). Top models for both spring and fall included a diagonal and unequal observation-error variance covariance matrix, indicating sampling variances for hunter population estimates that were spatially unique. Moreover, hunter population growth was variable among counties in spring ($\bar{u}$ = 0.04, *range* = -0.04–0.65 for *n* = 38 counties) and management units in fall ($\bar{u}$ = 0.12, *range* = -0.03–0.65 for *n* = 9 units; Fig 2; S4 Table).

**Table 2:** Results of model selection analyses to evaluate support for different hypothesized spatial scales of wild turkey hunter population dynamics for spring (Spring) and fall (Fall) hunting seasons in southern Michigan.

Model structure^a^				
Season and scale	Process error variance-covariance	Observation error variance-covariance	Δ AICc	*w*_*i*_
Spring				
County	Equal^b^	Diagonal-unequal^c^	0.00	1.00
Unit	Equal	Diagonal-unequal	299.32	0.00
Unit	Diagonal-unequal	Diagonal-unequal	316.90	0.00
Fall				
Unit	Equal	Diagonal-unequal	0.00	0.94
County	Equal	Diagonal-unequal	5.87	0.05
Unit	Diagonal-equal^d^	Diagonal-unequal	8.86	0.01

We ranked and compared models using Akaike’s Information Criterion corrected for small sample sizes (AIC_C_) and normalized Akaike model weights (*w*_*i*_).

^a^ Model structure reflected hypotheses that hunter population growth rates were unique at the county (County) or management unit (Unit) spatial scale, as well as hypotheses about the nature of variation and correlation of hunter population fluctuations over time (Process variance-covariance) and the variation and correlation of sampling errors among observations of turkey hunter populations (Observation variance-covariance).

^b^ Equal variance-covariance means that the magnitude of temporal variation of fluctuations (of hunter populations or sampling errors [not supported]) among sites is of equal magnitude, and that the correlation of such fluctuations among sites is shared among all pairs of sites. See S1 File for mathematical details.

^c^ Diagonal-unequal variance-covariance means that the magnitude of temporal variation of fluctuations (of hunter populations or sampling errors) is unique for each site, and that such fluctuations are independent among sites. See S1 File for mathematical details.

^d^ Diagonal-equal variance-covariance means that the magnitude of temporal variation of fluctuations (of hunter populations or sampling errors) among sites is of equal magnitude, and that such fluctuations are independent among sites. See S1 File for mathematical details.

Hunter population growth was also similarly structured in space for both spring and fall seasons, with a gradient of changing population growth moving from east to west (Fig 2). Specifically, the eastern portion of southern Michigan saw primarily positive growth in hunter populations, and also the strongest magnitude changes in hunter population growth over the study duration, yet they generally started from smaller populations of hunters (e.g., the county with the largest population growth rate [Wayne Co.] started with 0 spring hunters in 2001 and finished with 146 hunters in 2014). In contrast, negative hunter population growth was observed for many counties and units in the central and north-central portion of southern Michigan, whereas the western regions saw a mixture of positive and negative population growth (Fig 2). However, areas with negative growth rates often started from large populations of hunters and contracted in size over time (e.g., the county with the largest negative growth rate [Barry Co.] started with 2,752 spring hunters in 2001 and finished with 1,727 hunters in 2014).

Regression analyses suggested the factors known to provide opportunity for successful and high-quality turkey hunting had little meaningful effect on hunter populations in southern Michigan (S5 Table, S2 & S3 Figs). The negative binomial model parameterized with a linear variance-mean relationship was favored for both spring and fall seasons, as was the random effects structure with random intercepts and time trend slopes by county (S1 Table). In addition to a quadratic time-trend, the top spring model included effects of hunter interference, human population size, and density of male turkeys during spring hunting (S5 Table), yet all covariates were estimated imprecisely and had 95% confidence intervals that overlapped zero. Moreover, there was substantial model selection uncertainty (*w*_*i*_ = 0.14 for top model), including 6 models with ΔAIC_c_ ≤ 2 (S5 Table). Additional spring models that received support contained combinations of the above variables, as well as hunter success, area of public land open to hunting, and spring hunting season length covariates. However, none of the hypothesized variables had sizable effects on the expected number of spring hunters (S2 Fig). Two top fall models had effectively identical AIC_c_ scores and included, in addition to the quadratic time trends, effects of hunter success, human population size, and area of public land open to hunting (S5 Table). Again, covariate effects on hunter numbers were estimated imprecisely and had 95% confidence intervals that overlapped zero. Fall analyses also showed substantial model selection uncertainty (*w*_*i*_ = 0.09 for top models) and included 12 models with ΔAIC_c_ ≤ 2 (S5 Table). Additional fall models receiving support contained the previously mentioned variables, as well as turkey density and turkey population growth rates. Similar to spring models, however, none of the hypothesized variables had meaningful effects on fall hunter numbers (S3 Fig).

## Discussion

Dynamics of post-restoration turkey hunter effort were not uniform in space. Instead, we found heterogeneity at the scale of counties and management units within southern Michigan, as had previously been described over broader scales [10,11]. Further, we observed spatial structuring in the direction of hunter population growth among counties in spring, providing evidence for correlation of hunter numbers at intermediate scales (i.e., among counties) that did not necessarily correspond with existing management units. Importantly, we found little evidence that factors providing opportunity for successful and quality turkey hunting had meaningful impacts on turkey hunter numbers, at least not in recent years where hunter success and satisfaction metrics have remained relatively high. Our analyses thus highlight limitations of the current understanding of dynamics and drivers of turkey hunter populations and have implications for monitoring changes to hunter numbers as a component of existing harvest monitoring programs.

We demonstrate that heterogeneous patterns of hunter abundance can manifest themselves at a variety of spatial scales, not only the broad scales commonly described in the literature. Multiple studies have evaluated hunter population dynamics, as well as their contributing factors and policy implications, at statewide scales. For example, dynamics of deer hunter populations in Wisconsin suggested age, time period, and cohort effects on participation will likely result in fewer hunters in the future [8]. Statewide populations of deer and elk hunters were relatively stable over a recent 10-yr period in Montana, but also exhibited age, cohort, and gender-related differences in participation [30]. While these coarse-scale assessments may be sufficient for monitoring the broad-scale drivers and policy implications of changing hunter participation, our analyses demonstrate the existence of finer-scale structure to hunter population dynamics. Indeed, we found evidence that growth rates were specific to the county (spring) and management region (fall). These results are important because they imply a more thorough understanding of past hunter population changes, and importantly better prediction of future hunter populations, may be achieved by multi-scale analyses that incorporate spatial heterogeneity of population dynamics directly into analyses of past dynamics and projections of future participation.

The fine-scale heterogeneity of hunter participation we observed also has implications for state-level population monitoring programs for turkeys. Many states rely on raw harvest to index turkey population change through space and time [11], and fine-scale heterogeneity in hunter participation complicates interpretation of such data. Use of raw harvest to index turkey abundance in the absence of hunter effort data relies on assumptions that effort is unstructured in space and time (i.e., is constant or changes randomly among counties and years). Our results demonstrate that these assumptions are clearly false in southern Michigan. Specifically, turkey hunter populations are changing over time, the magnitude of this change is spatially structured at the county (spring) or management unit (fall) scale, and the directionality of change displays additional spatial structure along an east-west gradient in southern Michigan. Thus, use of raw harvest to index turkey abundance would result in inaccurate inferences about population trends even if other assumptions of the index were met [11,18,31]. Spatial-temporal changes to turkey harvest reflect changes to hunter participation in addition to changes in turkey abundance (e.g., S1 Fig). As such, the commonly made assumption of unstructured changes to turkey hunter effort appears untenable, making raw harvest a precarious monitoring metric for turkey populations, despite its widespread use. Given the fine- (this study) and broad-scale [10,11] heterogeneity in turkey hunter effort described in recent years, we believe it unlikely that effort patterns have remained spatially and temporally unstructured in other regions. If this assertion is correct, interpretation of either spatial or temporal changes to raw turkey harvest in areas that lack hunter effort data is difficult, as changes to harvest arise as a function of changes to populations of both hunters and turkeys.

While our results show that hunter populations fluctuated at scales finer than the ancestral range of turkeys in southern Michigan, we also found evidence for spatial structuring of hunter population change at intermediate scales. The magnitude of hunter population change was structured at scales finer than is commonly described, yet the directionality of hunter population change (+ or -) demonstrated an east-west regional pattern for both spring and fall hunting seasons. We expect the demographic structure of the hunter population (e.g., age, period, cohort effects) to affect turkey hunter dynamics at broad scales [8,30], and over the entire study area there is evidence that turkey hunter populations peaked during the middle 2000s but have since begun to decline (S1 Fig). However, it is not clear that demographic effects like hunter age structure or cohort effects would result in the spatial structuring of population change that we observed across southern Michigan. We would not expect the demographic structure of hunter populations to cause the observed spatial patterns unless the hunter demographics (e.g., age structure) were also spatially structured along a similar gradient, or unless the magnitude of age, period, or cohort effects themselves were heterogeneous in space and thus dependent on local or regional context. Moreover, while the causes of the observed east-west gradient remain unknown, we speculate this pattern could simply reflect the history of turkey restoration within the study area. For example, turkeys were first restored in the southwest portion of the study area, and this region therefore has the longest history of turkey hunting. In contrast, restoration of turkey populations (and hunting opportunities) was more recent in the eastern part of southern Michigan, which coincides with counties where positive growth of hunter populations was frequently observed.

Although hunting is an individual behavior, support structures and the social context under which hunting occurs (the so-called social habitat for hunting) may affect participation of individuals [32]. The social habitat for hunting consists of multiple hierarchically-nested levels of attributes that affect participation, including micro- (e.g., family and hunting mentors), meso- (e.g., community support networks), and macro-levels (e.g., demographics, urbanization), where factors at each level may constrain or facilitate hunting activities [32]. If micro-levels factors manifest themselves at more local spatial scales and meso- and macro-level factors manifest themselves at intermediate-broad scales, then the spatial scales of hunter population change that we observed would imply micro-level factors (e.g., family and hunting mentors) may be regulating the magnitude of spring turkey hunter effort at a local-scale in southern Michigan. Moreover, the east-west gradient of directionality in hunter population growth documented here would imply meso- or macro-level social factors may be spatially structuring those local-scale trends for turkey hunters across southern Michigan. We note, however, that the behavior of individuals was not the focus of this study. Consequently, it is possible that some of the spatial structure observed in hunter population change was caused by the spatial redistribution of hunters within Michigan over time (e.g., hunting closer to or further from one’s residence), rather than from individual hunters entering or leaving the total population of turkey hunters in southern Michigan. Also, given that restoration of turkey populations in the southeast portion of Michigan lagged behind recovery in the northern and western portion of the ancestral range, some hunters may have redistributed themselves during the timeframe of our study.

Regardless of the exact drivers of change in turkey hunter populations, our results imply that managers may have little ability to sizably affect participation in the future through traditional pathways like increasing opportunity for high-quality hunting. Our analyses provided little evidence that factors creating opportunity for quality hunting had sizable effects on spring and fall turkey hunter population at a county scale, at least not during recent periods where hunter success and satisfaction measures remain high (i.e., high quality hunting was normal). The motivations of individual hunters that affect their decision to hunt or not may be complex, but managers often measure satisfaction to assess the degree to which those motivations are fulfilled [5,33]. Yet, our analyses demonstrated that hunter satisfaction was in fact a poor predictor of hunter numbers at the county scale across southern Michigan. Similarly, hunter success in previous seasons had little effect on hunter numbers, which supports the notion that hunter motivations extend beyond opportunity to harvest (e.g., time outdoors with friends and family) [34]. This does not mean that hunter success and satisfaction are not important at all for hunter participation, and we suspect that consistently low measures of these metrics (i.e., lower than values observed in this study) would reduce hunting participation. In addition, we suspect that some of our estimated covariate effects may have limited ability to generalize to other regions. For instance, southern Michigan has very little public land, and the vast majority of turkey hunting activity occurs on private lands [19]. Thus, we suspect that availability of public hunting lands has more influence on the distribution of hunting effort in areas with more public access. Similarly, our measure of total season length had little contrast (6 days) and was complicated by the fact that different tag types are open for different periods during the season. Thus, our conclusion that season length had little bearing on participation may not hold in areas where a single general tag is available with open hunting during the entire season. Nonetheless, hunter participation across southern Michigan has clearly fluctuated in recent years (in both directions) irrespective of high hunt quality measures (S1 Fig), indicating just as clearly that other factors are influencing changes to hunter numbers through space and time.

Changes in game abundance would be expected to affect hunter participation at some level, for instance if game densities were low we would expect hunting participation to diminish [35,36]. Yet turkey hunter numbers in southern Michigan appear relatively unaffected by turkey abundance in recent years, which was also documented for turkey hunters in Missouri [12]. Turkey populations in southern Michigan appear stable to increasing, yet total hunter numbers across the region have begun to decline and participation patterns are clearly heterogeneous in space. Thus, similar to other species (e.g., deer and waterfowl) [7,37] there appears to be a breakdown of the historical relationship between abundance of turkey populations and the number of turkey hunters. This apparent decoupling of turkey abundance from hunter abundance creates a need to understand how changes to social values and the social context surrounding turkey hunting may have shifted over time [32,38].

While our results imply that managers may have limited ability to sizably increase hunter participation in the future through hunting quality alone, existing quota systems currently in place for managing turkeys in Michigan may affect the spatial distribution of hunters in any given year. For instance, if hunters want to pursue turkeys on public land in southern Michigan they must apply for and draw a license from the quota available in a given management unit (Fig 1A), whereas hunters that pursue turkeys on private lands are spatially unconstrained and can hunt in any management unit [19]. While it is possible such constraints could affect inferences about the effects of hunt quality metrics on the spatial-temporal distribution of hunters, we believe this was unlikely with our analyses. First, the vast majority of turkey hunters in southern Michigan (>80%) [19] purchase licenses that allow them to hunt on private lands only during spring, and thus their efforts are spatially unconstrained among counties within a given year. Indeed, >90% of turkeys harvested during spring in Michigan are harvested on private lands [19]. In addition, even though hunters who pursue turkeys on public lands are constrained to one management unit, the number of available licenses far exceed the number of applicants and license purchasers [19], which means hunters have the ability to self-select which general region they hunt in without constraint. Thus, turkey hunters in southern Michigan are not precluded from hunting their county of choice, and we believe our inferences about the limited utility of hunt quality metrics for explaining patterns of hunter participation are valid.

Turkeys are among the most popular game animals in the U.S. [4] and are likely to be an important component of the future of hunting as a sport. We demonstrate that hunter satisfaction does not explain ongoing changes to hunter populations, and that the opportunity for satisfactory hunting may not be enough to sustain turkey hunter numbers. The social context surrounding hunting can change over time [39], and thus the factors affecting turkey hunter participation now may not be the same as during the restoration period of management (i.e., pre 2000s) [2]. Whereas hunter populations increased in earlier time periods as turkey populations and hunting opportunities expanded [2,3], the novelty of abundant turkey populations may be wearing off, and large-scale demographic and social changes affecting other hunter groups may be finally catching up to turkey hunting. The spatially structured changes in hunter populations observed in this study do suggest at least the possibility of targeting recruitment and retention efforts in space, to strategically focus on localities with declining trends (e.g., north-central and western areas of southern Michigan). Yet the effectiveness of spatially targeted recruitment and retention efforts for sustaining or increasing local hunter populations remains untested. As such, additional research could test the effectiveness of spatially-targeted efforts, and also facilitate a better understanding of targeted recruitment and retention efforts relative to social factors operating in modern turkey management.

## Conclusions

Turkey hunter populations exhibit spatial heterogeneity in their dynamics, and recent variation in factors that result in quality turkey hunting appears to have had little impact on hunter numbers. Ongoing changes in turkey hunter participation in southern Michigan are not sizably affected by factors under direct management control, but are instead spatially structured within the region as a result of factors that are poorly understood, as large- and intermediate-scale factors affecting hunter support networks and the social context surrounding turkey hunting remain unstudied. Our results indicate the potential for spatially targeted recruitment and retention efforts focused on localities with declining participation, but also suggest such efforts would be most effective if informed by additional human dimensions research to better understand the current social context for turkey hunting. Lastly, heterogeneity in the direction and magnitude of hunter population trajectories suggests that monitoring programs relying on raw harvest for indexing turkey populations are providing ambiguous information about changes in those populations.

## Supporting information

S1 File MARSS modeling details. (PDF)

S1 Fig Estimated total turkey hunter population size, raw harvest, and harvest-per-unit effort during spring hunting seasons in southern Michigan, USA (2001–2014). (PDF)

S2 Fig Model-averaged prediction plots of expected number of spring turkey hunters as a function of covariates in the top mixed-effect negative binomial regression models describing the relationships between covariates and county-scale wild turkey hunter numbers in southern Michigan, USA. (PDF)

S3 Fig Model-averaged prediction plots of expected number of fall turkey hunters as a function of covariates in the top mixed-effect negative binomial regression models describing the relationships between covariates and county-scale wild turkey hunter numbers in southern Michigan, USA. (PDF)

S1 Table Results of model-selection analyses to evaluate random-effects structures and statistical distributions for modeling drivers of estimated county-scale wild turkey hunter population size during spring and fall hunting seasons in southern Michigan, USA. (PDF)

S2 Table Results of preliminary assessment of effects of hunter perception and turkey population dynamics covariates on estimated county-scale spring turkey hunter population size in southern Michigan, USA (2001–2014). (PDF)

S3 Table Results of preliminary assessment of effects of hunter perception and turkey population dynamics covariates on estimated county-scale fall turkey hunter population size in southern Michigan, USA (2002–2013). (PDF)

S4 Table Estimated county-scale annual growth rates (natural log scale) for spring and fall hunting seasons for turkey hunter populations in southern Michigan, USA. (PDF)

S5 Table Results of model selection analyses to evaluate support for hypothesized drivers of county-scale wild turkey hunter population size during spring and fall hunting seasons in southern Michigan, USA. (PDF)

## References

[1] Bailey RW. The wild turkey status and outlook in 1979. Proc of the National Wild Turkey Symposium. 1980:4: 1–9.

[2] Lewis JB. A success story revisited. Proc of the National Wild Turkey Symposium 2001:8: 7–13.

[3] Tapley JL, Abernathy RK, Kennamer JE. Status and distribution of the wild turkey in 2004. Proc of the National Wild Turkey Symposium 2007:9: 21–31.

[4] Harris A. Turkey hunting in 2006: an analysis of hunter demographics, trends, and economic impacts: addendum to the 2006 national survey of fishing, hunting, and wildlife-associated recreation. Arlington, Virginia: US Fish and Wildlife Service Report; 2010.

[5] Enck JW, Decker DJ, Brown TL. Status of hunter recruitment and retention in the United States. Wild Society Bulletin 2000:28: 817–824.

[6] Porter WF, Healy WM, Backs SE, Wakeling BF, Steffen DE. Managing turkeys in the face of uncertainty. Proc of the National Wild Turkey Symposium 2011:10: 1–9.

[7] Vrtiska MP, Gammonley JH, Naylor LW, Raedeke AH. Economic and conservation ramifications from the decline of waterfowl hunters. Wild Soc Bulletin 2013:37: 380–388.

[8] Winkler R, Warnke K. The future of hunting: an age-period-cohort analysis of deer hunter decline. Population and Environment 2013:34: 460–480.

[9] Tapley JL, Healy WM, Abernathy RK, Kennamer JE. Status of wild turkey hunting in North America. Proc of the National Wild Turkey Symposium 2001:8: 15–22.

[10] Ericksen RE, Hughes TW, Brown TA, Akridge MD, Scott KB, Penner CS. Status and distribution of wild turkeys in the United States: 2014 status. Proc of the National Wild Turkey Symposium 2016:11: 7–18.

[11] Parent CJ, Stevens BS, Bowling AC, Porter WF. Wild turkey harvest trends across the Midwest in the 21st century. Proc of the National Wild Turkey Symposium 2016:11: 211–223.

[12] Clawson MV, Skalski JR, Isabelle JL, Millspaugh JJ. Trends in male wild turkey abundance and harvest following restoration efforts in the southeast regions of Missouri, 1960–2010. Wild Soc Bulletin 2015:39: 116–128.

[13] Hawn LJ, Langenau EE, Reis TF. Optimization of quantity and quality of turkey hunting in Michigan. Wild Soc Bulletin 1987:15: 233–238.

[14] Cartwright ME, Smith RA. Attitudes, opinions, and characteristics of a select group of Arkansas spring turkey hunters. Proc of the National Wild Turkey Symposium 1990:6: 177–187.

[15] Little DA, Bowman JL, Hurst GA, Seiss RS, Minnis DL. Evaluating turkey hunter attitudes on wildlife management areas in Mississippi. Proc of the National Wild Turkey Symposium 2000:8: 223–231.

[16] Swanson DA, Stoll RJ, Culbertson WL. Attitudes, preferences, and characteristics of Ohio’s spring turkey hunters, 1985–2001. Proc of the National Wild Turkey Symposium 2007:9: 325–330.

[17] Isabelle JL, Reitz RA. Characteristics, attitudes, and preferences of spring wild turkey hunters in Missouri. Proc of the National Wild Turkey Symposium 2016:11: 249–258.

[18] Maunder MN, Sibert JR, Fonteneau A, Hampton J, Kleiber P, Harley SJ. Interpreting catch per unit effort data to assess the status of individual stocks and communities. ICES Journal of Marine Science 2006:63: 1373–1385.

[19] Frawley BJ, Boone CE. 2014 Michigan spring turkey hunter survey Lansing, Michigan: Wildlife Division Report No. 3607, Michigan Department of Natural Resources; 2015.

[20] Frawley BJ. 2013 Michigan spring turkey hunter survey. Lansing, Michigan: Wildlife Division Report No. 3579, Michigan Department of Natural Resources; 2014.

[21] Frawley BJ. 2013 Michigan fall turkey hunter survey. Lansing, Michigan: Wildlife Division Report No. 3392, Michigan Department of Natural Resources; 2014.

[22] Holmes EE, Ward EJ, Scheuerell MD. Analysis of multivariate time-series using the MARSS package: version 3.9 Seattle, Washington: NOAA Fisheries, Northwest Fisheries Science Center; 2014.

[23] Burnham KP, Anderson DR. Model selection and multimodel inference: a practical information theoretic approach 2nd ed. New York, New York: Springer-Verlag; 2002.

[24] Holmes EE, Derivation of the EM algorithm for constrained and unconstrained marss models Seattle Washington: Technical report, Northwest Fisheries Science Center; 2012.

[25] O’Hara RB, Kotze DJ. Do not log transform count data. Meth in Ecol and Evol 2010:1: 118–122.

[26] Hilbe JM. Negative binomial regression Cambridge, UK: Cambridge University Press; 2011.

[27] Irwin BJ, Wagner T, Bence JR, Kepler MV, Liu W, Hayes DB. Estimating spatial and temporal components of variation in fisheries count data using negative binomial mixed models. Trans of the Amer Fisheries Soc 2013:142: 171–183.

[28] Fournier DA, Skaug HJ, Ancheta J, Ianelli J, Magnusson A, Maunder MN, et al AD Model Builder: using automatic differentiation for statistical inference of highly parameterized complex nonlinear models. Optimization Meth and Software 2012:27: 233–249.

[29] Skaug HD, Fournier D, Nielson A, Magnusson A, Bolker B. glmmADMB: generalized linear mixed models using ADMB. 2011. R package version 0.6.

[30] Schorr RA, Lukacs PM, Gude JA. The Montana deer and elk hunting population: the importance of cohort group, license price, and population demographics on hunter retention, recruitment, and population change. J. of Wild Man 2014:78: 944–952.

[31] Hilborn R, Walters CJ. Quantitative fisheries stock assessment: choice, dynamics and uncertainty Dordecht, Netherlands: Springer Science + Business Media; 1992.

[32] Larson LR, Stedman RC, Decker DJ, Siemer WF, Baumer MS. Exploring the social habitat for hunting: toward a comprehensive framework for understanding hunter recruitment and retention. Human Dim of Wild 2014:19: 105–122.

[33] Manfredo MJ, Vaske JJ, Decker DJ. Human dimensions of wildlife management: basic concepts In Knight RL, Gutzwiller KJ, editors. Wildlife and recreationists: coexistence through management and research. Washington DC: Island Press; 1995 pp 17–31.

[34] Ryan EL, Shaw B. Improving hunter recruitment and retention. Human Dim of Wildlife 2011:16: 311–317.

[35] Miller JR. Game availability and hunter participation: a study of Washington elk hunting. The Annals of Regional Science 1982:16: 79–94.

[36] Miller CA, Vaske JJ. Individual and situational influences on declining hunter effort in Illinois. Human Dim of Wildlife 2003:8: 263–276.

[37] Riley SJ, Decker DJ, Enck JW, Curtis PD, Lauber TB, Brown TL. Deer populations up, hunter populations down: implications of interdependence of deer and hunter population dynamics on management. Ecoscience 2003:10: 455–461.

[38] Manfredo MJ, Teel TL, Bright AD. Why are public values toward wildlife changing? Human Dim of Wildlife 2003:8: 287–306.

[39] Enck JW, Swift BL, Decker DJ. Reasons for decline in duck hunting: insights from New York. Wild Soc Bulletin 1993:21: 10–21.

