# Supplementary material for: Spatial-temporal dynamics of hunter effort for wild turkeys in Michigan
Source: PLoS One. 2020 Apr 1;15(4):e0230747. doi: 10.1371/journal.pone.0230747 (PMC7112203; doi:10.1371/journal.pone.0230747)
Supplement: S1 File — (PDF) [file pone.0230747.s001.pdf]

File S1. MARSS modeling details.

We built multivariate autoregressive state-space models (MARSS models) to describe turkey hunter population change, and the basic approach was described in the Methods section (additional details and examples are found in Holmes et al. [2014], chapter 7). These models facilitated linking observed estimates of participating hunters (county scale) to model parameters for estimating population growth rates and the scales of population change (either county, management unit, or region-wide scale): In matrix form, the mathematical model structure is

$$\boldsymbol{x_t} = \boldsymbol{x_{t-1}} + \boldsymbol{u} + \boldsymbol{w_t}$$

$$\boldsymbol{w_t} \sim MVN(0, \boldsymbol{Q})$$

$$\boldsymbol{y_t} = \boldsymbol{Z}\boldsymbol{x_t} + \boldsymbol{a} + \boldsymbol{v_t}$$

$$\boldsymbol{v_t} \sim MVN(0, \boldsymbol{R})$$

$$\boldsymbol{x_0} \sim MVN(\boldsymbol{\pi}, \boldsymbol{\Lambda}),$$

where $\boldsymbol{x_t}$ was a $p \times 1$ column vector containing the log scale abundance for each of p latent hunter populations (p = m = number of counties, or number of management units, or 1 [for the entire region as 1 population]) in year t (t =1,…,T years), $\boldsymbol{y_t}$ was a $m \times 1$ column vector containing the county-level estimates of participating hunters in year t (i.e., the observed data on the log scale), stochastic process errors in population growth in year t were contained in $p \times 1$ column vector $\boldsymbol{w_t}$, and time invariant population growth rates were contained in the $p \times 1$ column vector $\boldsymbol{u}$. Estimated numbers of participating hunters for each county and year were linked to true hunter populations through the $m \times p$ matrix $\boldsymbol{Z}$ that specifies to which of the p populations each observed county-level time series are associated (analogous to a design matrix containing 0s and 1s to indicate which counties belong to which populations). In addition,

estimated numbers of participating hunters in each county are further linked to underlying true hunter populations by the $m \times 1$ column vector $\boldsymbol{a}$ (scaling coefficients representing the fraction of the underlying population that overlapped with each county, where estimates of participating hunters were generated at the county scale), and the realized observation errors contained in the time-specific $m \times 1$ column vector $\boldsymbol{v_t}$. Variance-covariance matrices specifying the variance and covariance for realized process and observation errors were specified via the matrices $\boldsymbol{Q}$ $(p \times p)$ and $\boldsymbol{R}$ $(m \times m)$, and $\boldsymbol{x_0}$ was a $p \times 1$ column vector of the initial hunter population sizes, whereas $\boldsymbol{\pi}$ and $\boldsymbol{\Lambda}$ are the vector of mean abundances and the variance-covariance matrix for the initial abundances.

We specified three plausible scales of change for hunter populations, and specified models representing each hypothesized scale where the underlying populations were linked back to county-level estimates of participating hunters via constraints on process and observation model matrices $\boldsymbol{u}$ and $\boldsymbol{Z}$. Here we provide details of how changes to $\boldsymbol{u}$ and $\boldsymbol{Z}$ specified models representing each hypothesis for the models of spring hunter dynamics. Models of fall hunter dynamics were specified analogously. First, we hypothesized that hunter populations fluctuated at the county scale (i.e., each county was a unique population with a unique growth rate), which corresponds to

$$\begin{bmatrix} x_1 \\ x_2 \\ \vdots \\ x_{37} \\ x_{38} \end{bmatrix}_t = \begin{bmatrix} x_1 \\ x_2 \\ \vdots \\ x_{37} \\ x_{38} \end{bmatrix}_{t-1} + \begin{bmatrix} u_1 \\ u_2 \\ \vdots \\ u_{37} \\ u_{38} \end{bmatrix} + \begin{bmatrix} w \\ w_2 \\ \vdots \\ w_{37} \\ w_{38} \end{bmatrix}_t,$$

and an observation model linking the 38 county-level estimates to the true population, $\boldsymbol{y_t} = \boldsymbol{Z x_t} + \boldsymbol{a} + \boldsymbol{v_t}$,

$$\begin{bmatrix} y_1 \\ y_2 \\ \vdots \\ y_{37} \\ y_{38} \end{bmatrix}_t = \begin{bmatrix} 1 & 0 & \dots & \dots & 0 & 0 \\ 0 & 1 & \ddots & & & 0 \\ \vdots & \ddots & \ddots & \ddots & & \vdots \\ \vdots & & \ddots & \ddots & \ddots & \vdots \\ 0 & & & \ddots & 1 & 0 \\ 0 & 0 & \dots & \dots & 0 & 1 \end{bmatrix} \begin{bmatrix} x_1 \\ x_2 \\ \vdots \\ x_{37} \\ x_{38} \end{bmatrix}_t + \begin{bmatrix} v_1 \\ v_2 \\ \vdots \\ v_{37} \\ v_{38} \end{bmatrix}_t ,$$

with a 38×38 diagonal matrix for **Z**. For all modeling we used the default "scaling" setting for **a**, and for this case where each county is a unique population, the ***y***s are direct estimates of the total ***x***s, and thus **a** is dropped as it is not estimated and is assumed to be a vector of zeros (see Holmes et al. [2014] for additional details).

Second, we hypothesized hunter populations fluctuated at the management unit scale (i.e., each management unit was a population and counties within management units had shared growth rates), which corresponds to

$$\begin{bmatrix} x_1 \\ x_2 \\ x_3 \\ x_4 \\ x_5 \\ x_6 \end{bmatrix}_t = \begin{bmatrix} x_1 \\ x_2 \\ x_3 \\ x_4 \\ x_5 \\ x_6 \end{bmatrix}_{t-1} + \begin{bmatrix} u_1 \\ u_2 \\ u_3 \\ u_4 \\ u_5 \\ u_6 \end{bmatrix} + \begin{bmatrix} w_1 \\ w_2 \\ w_3 \\ w_4 \\ w_5 \\ w_6 \end{bmatrix}_t ,$$

and observation model

$$\begin{bmatrix} y_1 \\ y_2 \\ \vdots \\ y_{37} \\ y_{38} \end{bmatrix}_t = \begin{bmatrix} 1 & 0 & 0 & 0 & 0 & 0 \\ \vdots & \vdots & \vdots & \vdots & \vdots & \vdots \\ 1 & 0 & 0 & 0 & 0 & 0 \\ 0 & 1 & 0 & 0 & 0 & 0 \\ \vdots & \vdots & \vdots & \vdots & \vdots & \vdots \\ 0 & 1 & 0 & 0 & 0 & 0 \\ 0 & 0 & 1 & 0 & 0 & 0 \\ \vdots & \vdots & \vdots & \vdots & \vdots & \vdots \\ 0 & 0 & 1 & 0 & 0 & 0 \\ 0 & 0 & 0 & 1 & 0 & 0 \\ \vdots & \vdots & \vdots & \vdots & \vdots & \vdots \\ 0 & 0 & 0 & 1 & 0 & 0 \\ 0 & 0 & 0 & 0 & 1 & 0 \\ \vdots & \vdots & \vdots & \vdots & \vdots & \vdots \\ 0 & 0 & 0 & 0 & 1 & 0 \\ 0 & 0 & 0 & 0 & 0 & 1 \\ \vdots & \vdots & \vdots & \vdots & \vdots & \vdots \\ 0 & 0 & 0 & 0 & 0 & 1 \end{bmatrix} \begin{bmatrix} x_1 \\ x_2 \\ x_3 \\ x_4 \\ x_5 \\ x_6 \end{bmatrix}_t + \begin{bmatrix} 0 \\ a_2 \\ \vdots \\ a_{37} \\ a_{38} \end{bmatrix} + \begin{bmatrix} v_1 \\ v_2 \\ \vdots \\ v_{37} \\ v_{38} \end{bmatrix}_t .$$

with a 38×6 matrix for **Z** linking the estimates of participating hunters for the 38 counties to the 6 management region-level hunter populations. In this case (and below) where the number of observed counties is not equal to the number of true populations, a is included but effectively represents a vector of nuisance parameters that are not a focus of inference.

Lastly, we hypothesized that there was a single population of turkey hunters across the study area (i.e., a single growth rate) and thus county-level estimates of participating hunters are merely replicated observations of the same underlying population process, which corresponds to a population model of

$$x_t = x_{t-1} + u + w_t$$

$$w_t \sim Normal(0, q),$$

and observation model

$$\begin{bmatrix} y_1 \\ y_2 \\ \vdots \\ y_{37} \\ y_{38} \end{bmatrix}_t = \begin{bmatrix} 1 \\ 1 \\ \vdots \\ 1 \\ 1 \end{bmatrix} x_t + \begin{bmatrix} 0 \\ a_2 \\ \vdots \\ a_{37} \\ a_{38} \end{bmatrix} + \begin{bmatrix} v_1 \\ v_2 \\ \vdots \\ v_{37} \\ v_{38} \end{bmatrix}_t .$$

In addition, the variance-covariance structures for the multivariate normal process and observation errors were specified through the matrices $\boldsymbol{Q}$ and $\boldsymbol{R}$. We considered four plausible process error variance-covariance structures for $\boldsymbol{Q}$, demonstrated below with a 3×3 matrix for simplicity (where q = process-error variance and $c_{i,j}$ = process-error covariance between populations i and j and $c_{i,j} = c_{j,i}$), including unconstrained

$$\begin{bmatrix} q_1 & c_{1,2} & c_{1,3} \\ c_{2,1} & q_2 & c_{2,3} \\ c_{3,1} & c_{3,2} & q_3 \end{bmatrix},$$

equal

$$\begin{bmatrix} q & c & c \\ c & q & c \\ c & c & q \end{bmatrix},$$

diagonal and unequal

$$\begin{bmatrix} q_1 & 0 & 0 \\ 0 & q_2 & 0 \\ 0 & 0 & q_3 \end{bmatrix},$$

and diagonal and equal variance-covariance structures

$$\begin{bmatrix} q & 0 & 0 \\ 0 & q & 0 \\ 0 & 0 & q \end{bmatrix}.$$

Note that the actual dimension of $\boldsymbol{Q}$ were $p \times p$ and therefore varied with the hypothesized population model structure. We also considered the diagonal and equal, and diagonal and unequal parameterizations shown above for $\boldsymbol{R}$. However, the observation-error variance-covariance matrices were all $m \times m$, as the number of counties (m) with observed time series of estimated hunter participation did not change among models within a given hunting season.

LITERATURE CITED

Holmes, E. E., E. J. Ward and M. D. Scheuerell. 2014. Analysis of multivariate time-series using the MARSS package: version 3.9. NOAA Fisheries, Northwest Fisheries Science Center, Seattle, Washington, USA.
