# Supplementary material for: Spatial-temporal dynamics of hunter effort for wild turkeys in Michigan
Source: PLoS One. 2020 Apr 1;15(4):e0230747. doi: 10.1371/journal.pone.0230747 (PMC7112203; doi:10.1371/journal.pone.0230747)
Supplement: S1 Fig — (PDF) [file pone.0230747.s002.pdf]

Figure S1. Estimated total turkey hunter population size (A), raw harvest (B), and harvest-per-unit effort (C) during spring hunting seasons in southern Michigan, USA (2001-2014).

A)

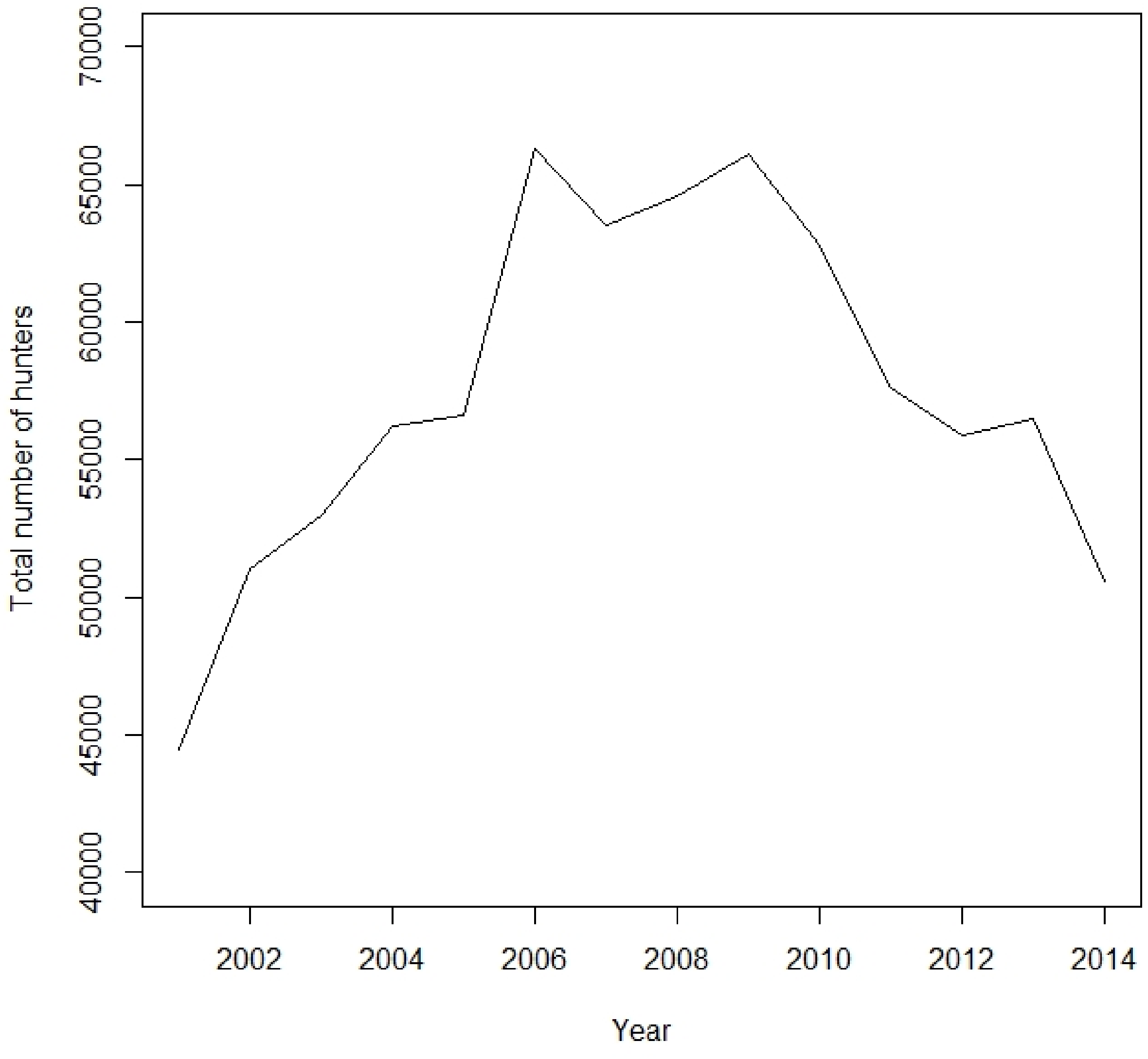

B)

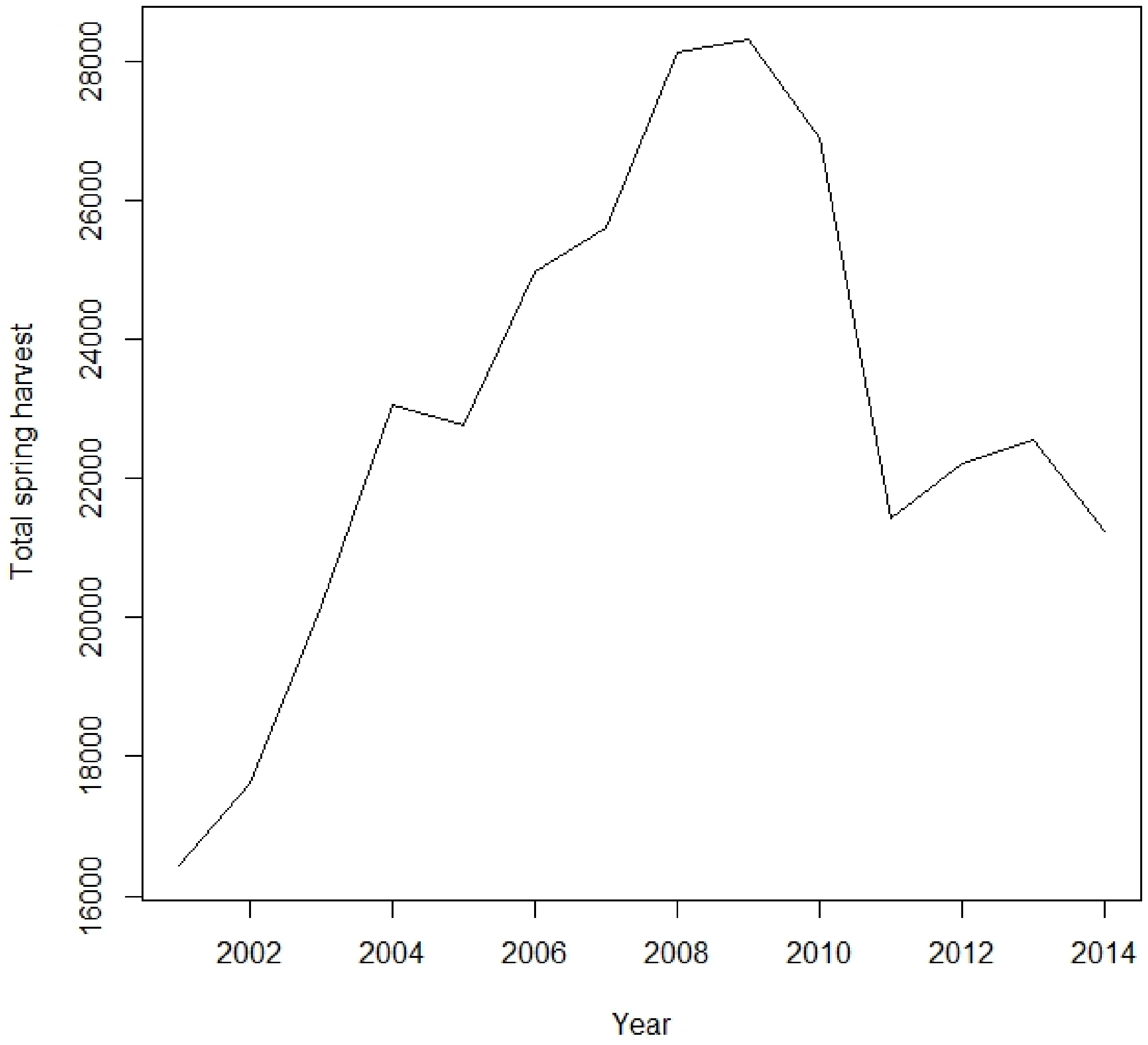

Total spring harvest
16000
18000
20000
22000
24000
26000
28000
2002
2004
2006
2008
2010
2012
2014
Year

C)

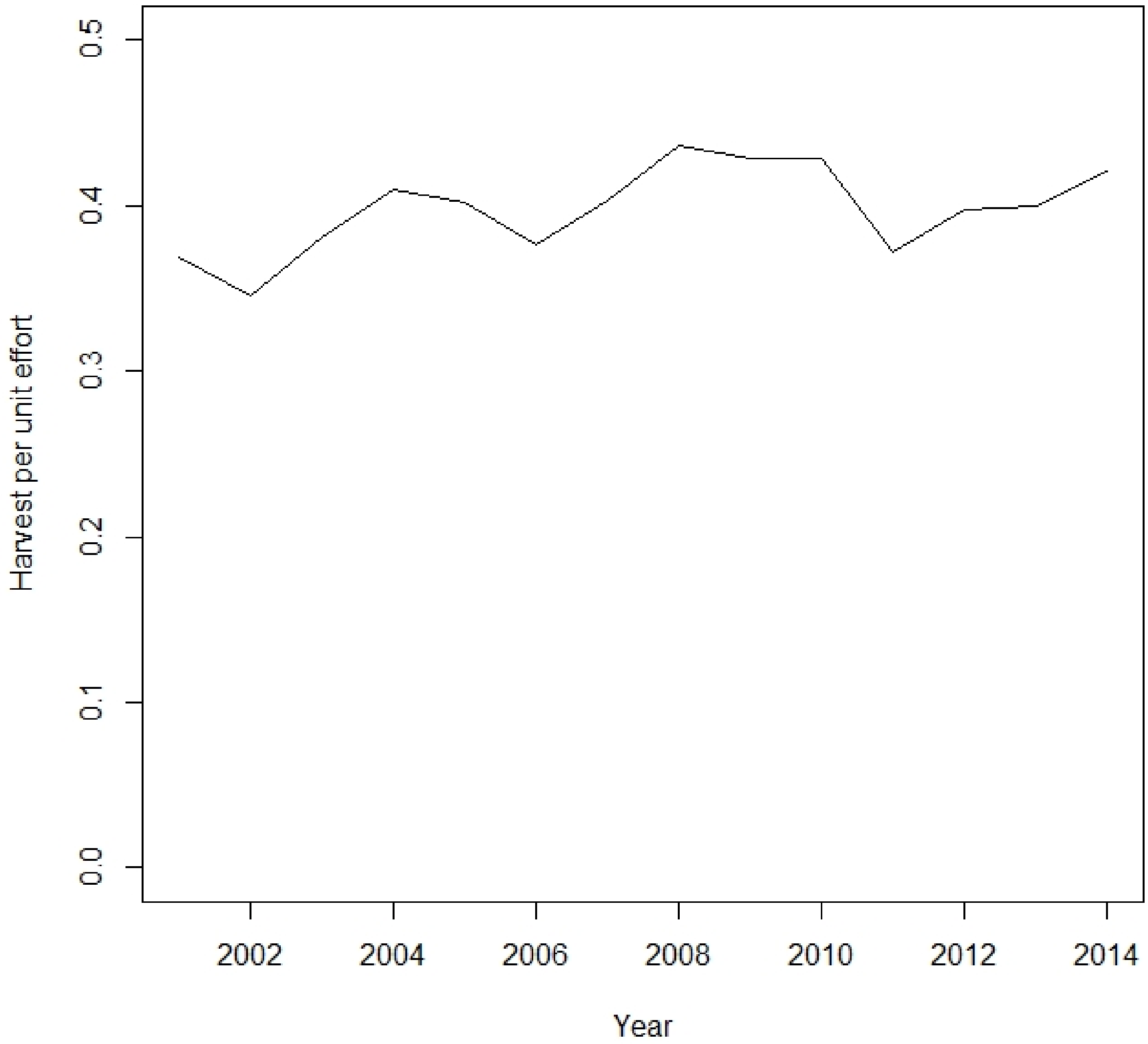
0.0
0.1
0.2
0.3
0.4
0.5
Harvest per unit effort
2002
2004
2006
2008
2010
2012
2014
Year
