# Supplementary material for: Spatial-temporal dynamics of hunter effort for wild turkeys in Michigan
Source: PLoS One. 2020 Apr 1;15(4):e0230747. doi: 10.1371/journal.pone.0230747 (PMC7112203; doi:10.1371/journal.pone.0230747)
Supplement: S2 Fig — (PDF) [file pone.0230747.s003.pdf]

Figure S2. Model-averaged prediction plots of expected number of spring turkey hunters as a function of covariates in the top mixed-effect negative binomial regression models describing the relationships between covariates and county-scale wild turkey hunter numbers in southern Michigan, USA.

A)

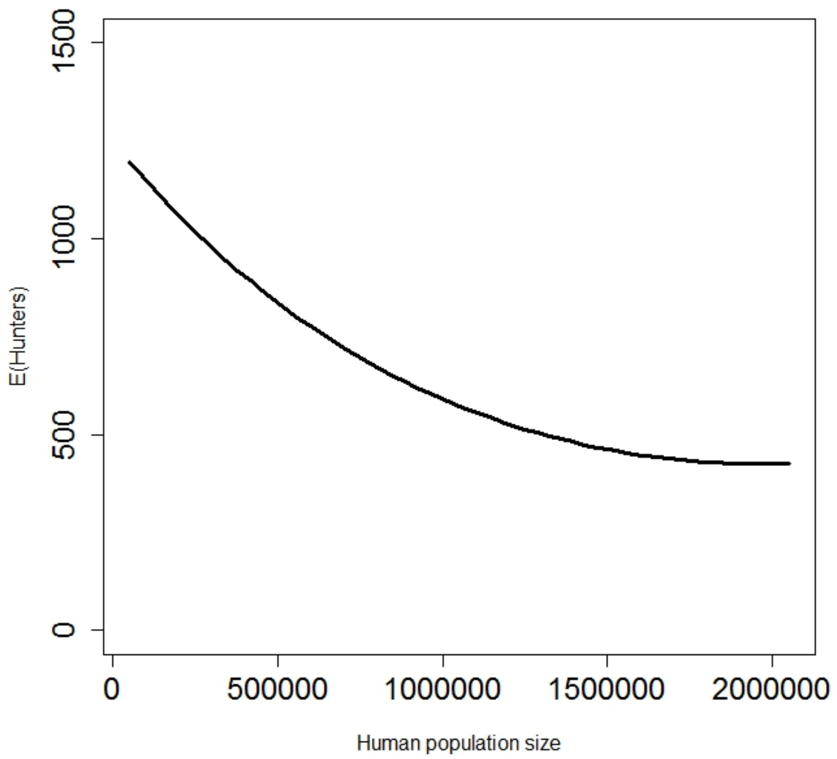

B)

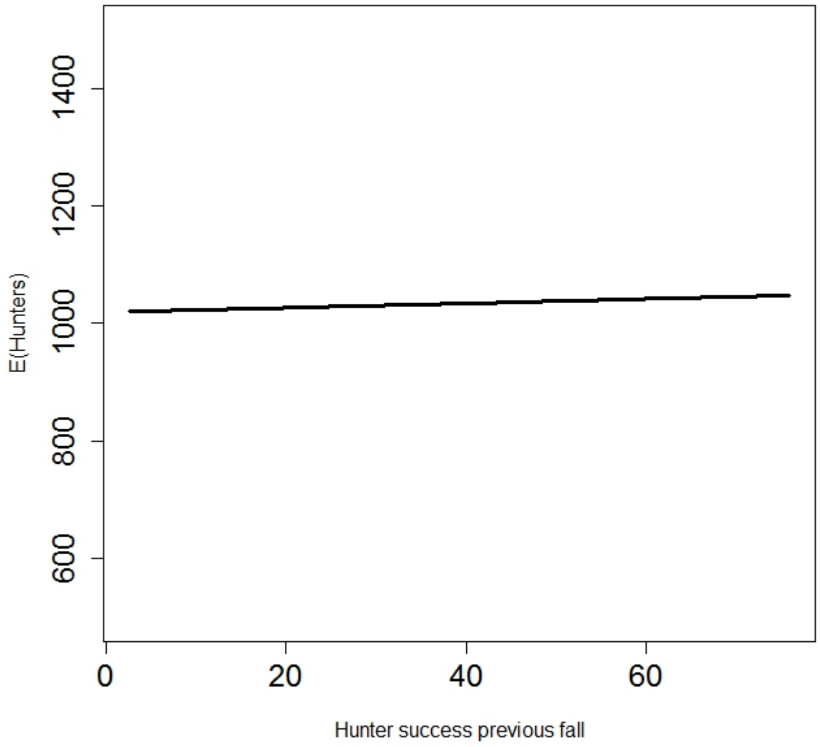

E(Hunters)
600
800
1000
1200
1400
0
20
40
60
Hunter success previous fall

C)

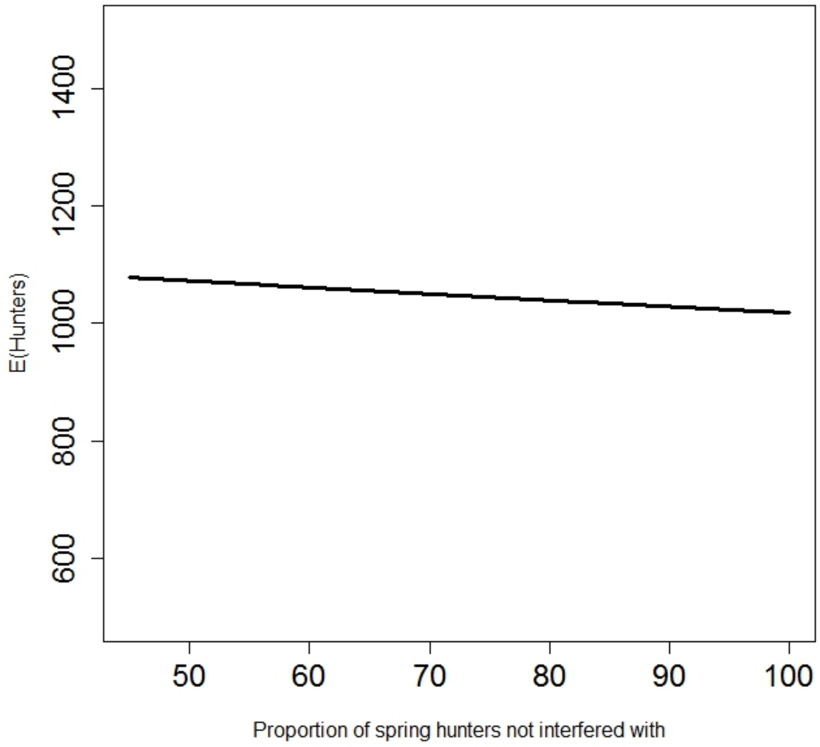
E(Hunters)
600
800
1000
1200
1400
50
60
70
80
90
100
Proportion of spring hunters not interfered with

D)

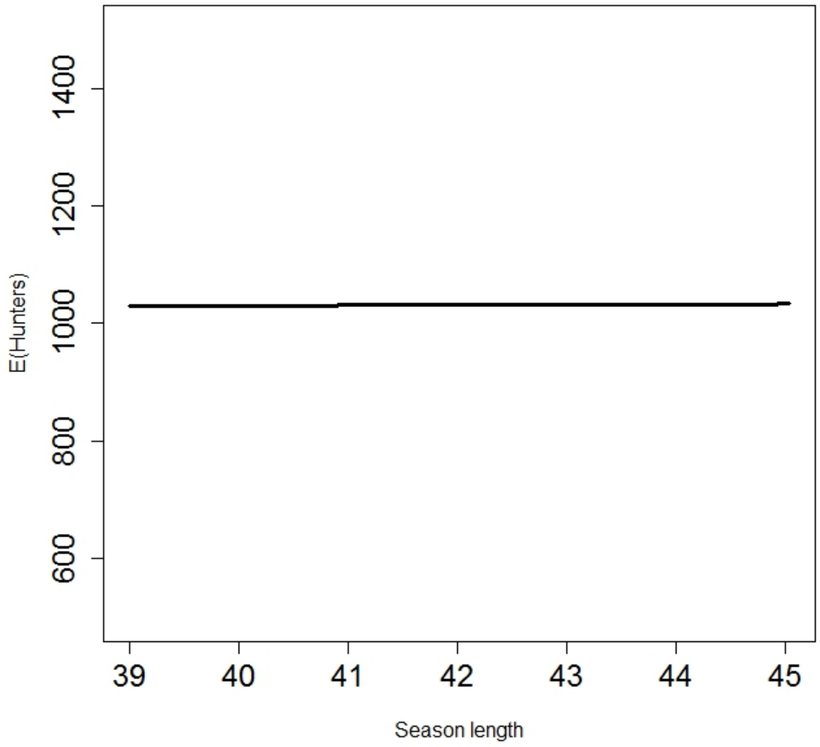

E(Hunters)
600
800
1000
1200
1400
39
40
41
42
43
44
45
Season length

E)

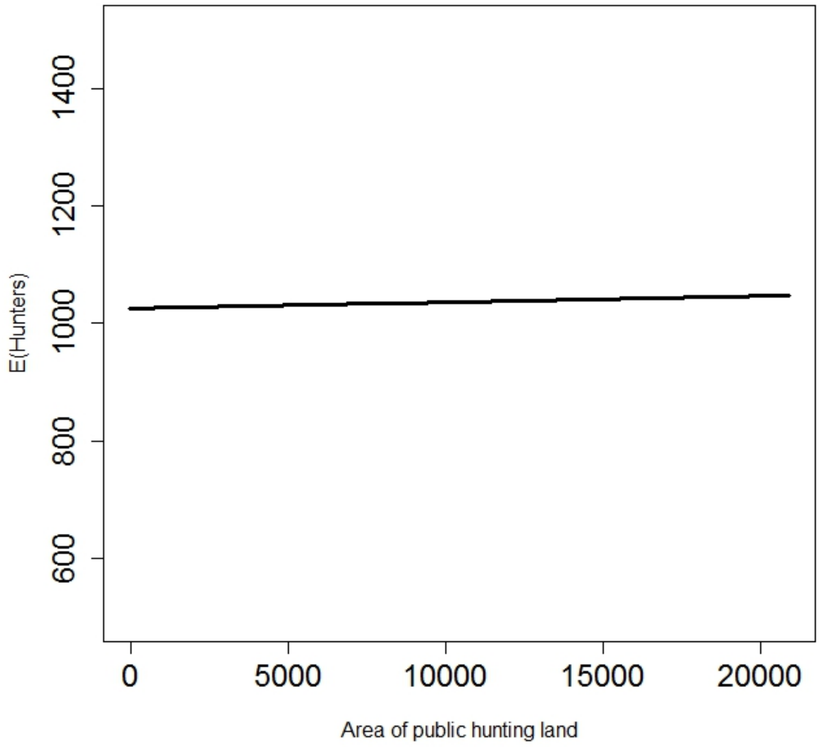

1400
1200
1000
800
600
E(Hunters)
0
5000
10000
15000
20000
Area of public hunting land

F)

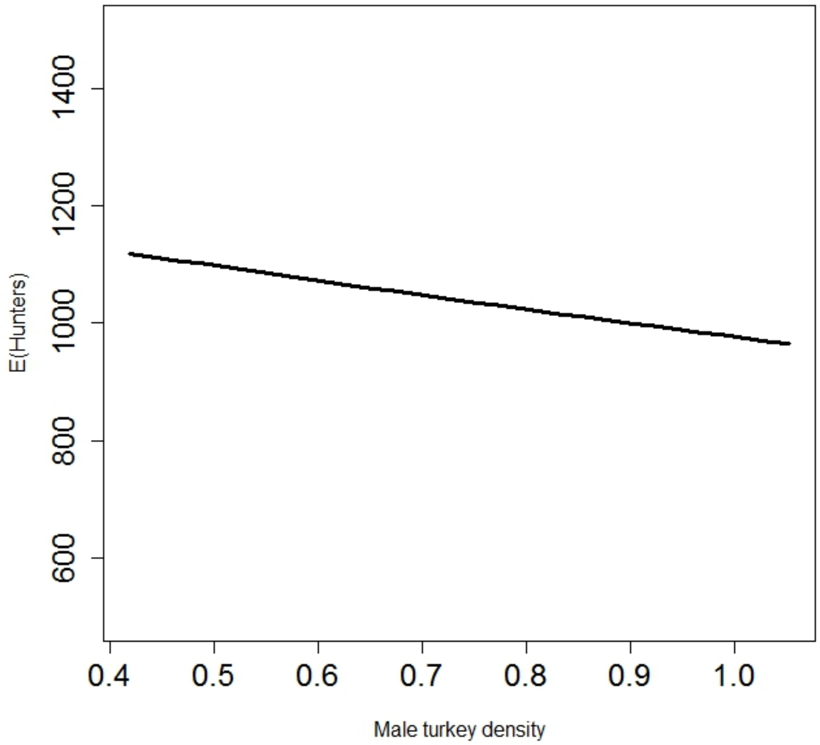

Expected number of hunters (E(Hunters)) was modeled as a function of the county-scale covariates human population size (A), proportion of successful hunters during the previous fall hunting season (B), proportion of hunters in the previous years' spring hunting season that were not interfered with (C), the current years' spring hunting season length in days (D), and the area of public lands open to hunting (E), as well as the management-unit scale covariate for density of male turkeys at the start of the hunting season during the previous spring.
