# Supplementary material for: Spatial-temporal dynamics of hunter effort for wild turkeys in Michigan
Source: PLoS One. 2020 Apr 1;15(4):e0230747. doi: 10.1371/journal.pone.0230747 (PMC7112203; doi:10.1371/journal.pone.0230747)
Supplement: S3 Fig — (PDF) [file pone.0230747.s004.pdf]

Figure S3. Model-averaged prediction plots of expected number of fall turkey hunters as a function of covariates in the top mixed-effect negative binomial regression models describing the relationships between covariates and county-scale wild turkey hunter numbers in southern Michigan, USA.

A)

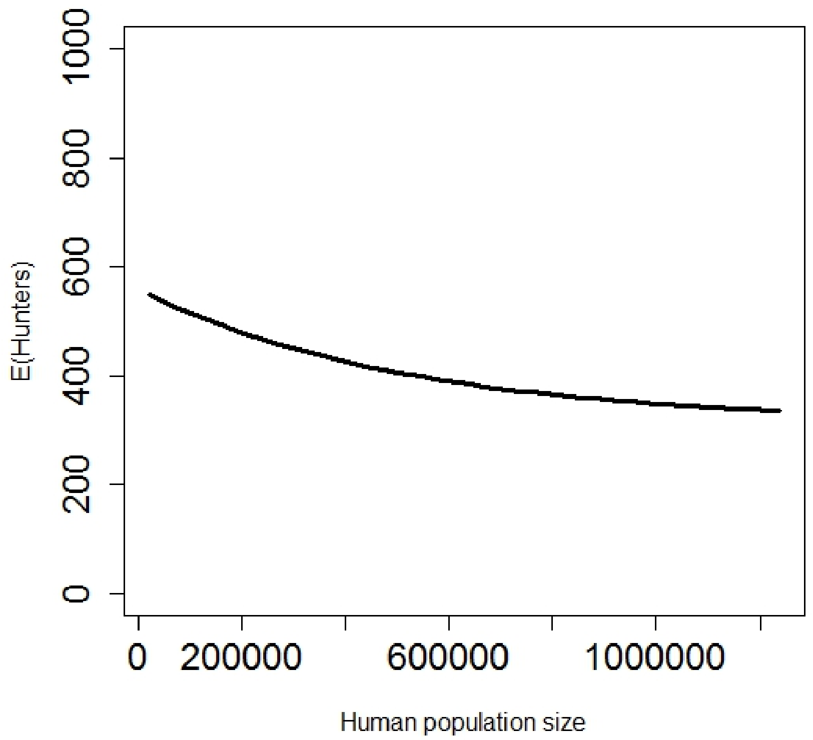

B)

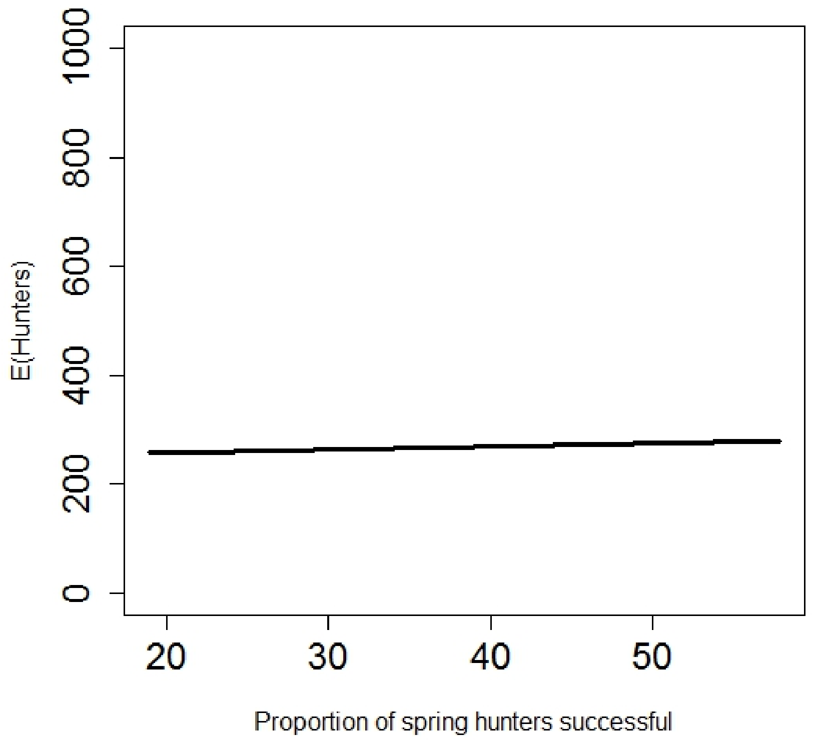
E(Hunters)
0
200
400
600
800
1000
20
30
40
50
Proportion of spring hunters successful

C)

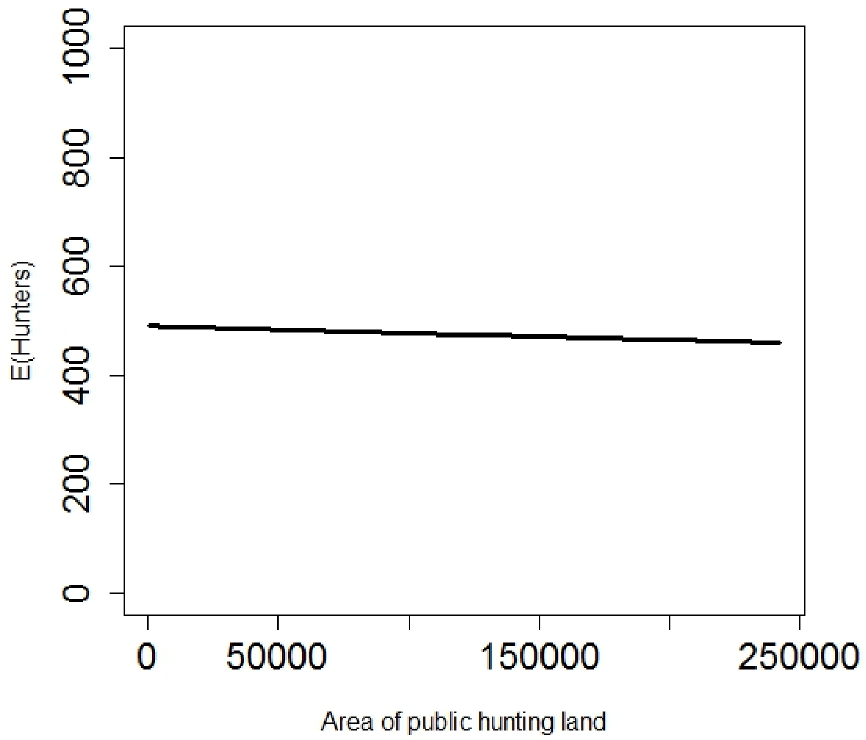

E(Hunters)
0
200
400
600
800
1000
0
50000
150000
250000
Area of public hunting land

D)

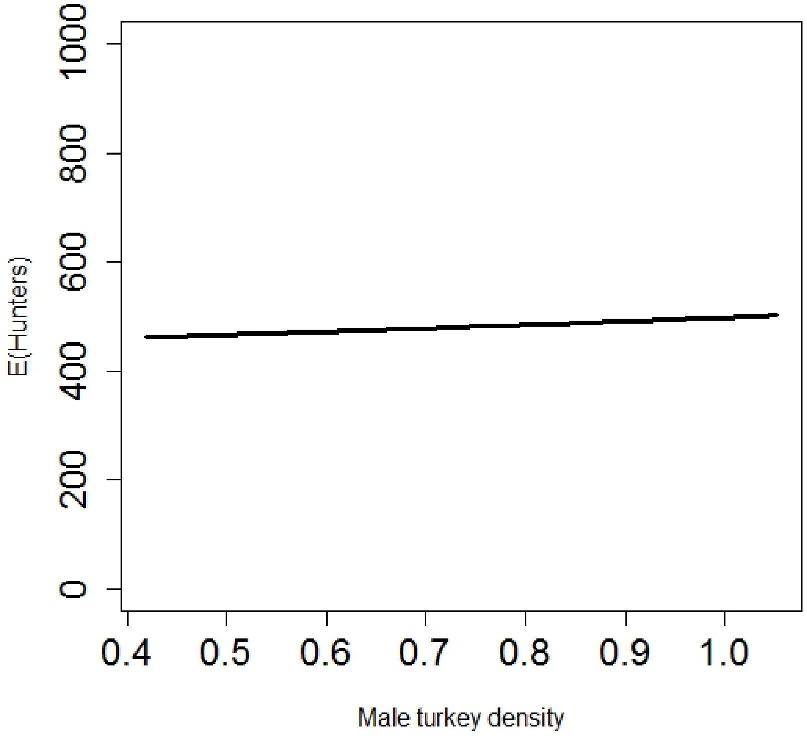

E(Hunters)
0
200
400
600
800
1000
0.4
0.5
0.6
0.7
0.8
0.9
1.0
Male turkey density

E)

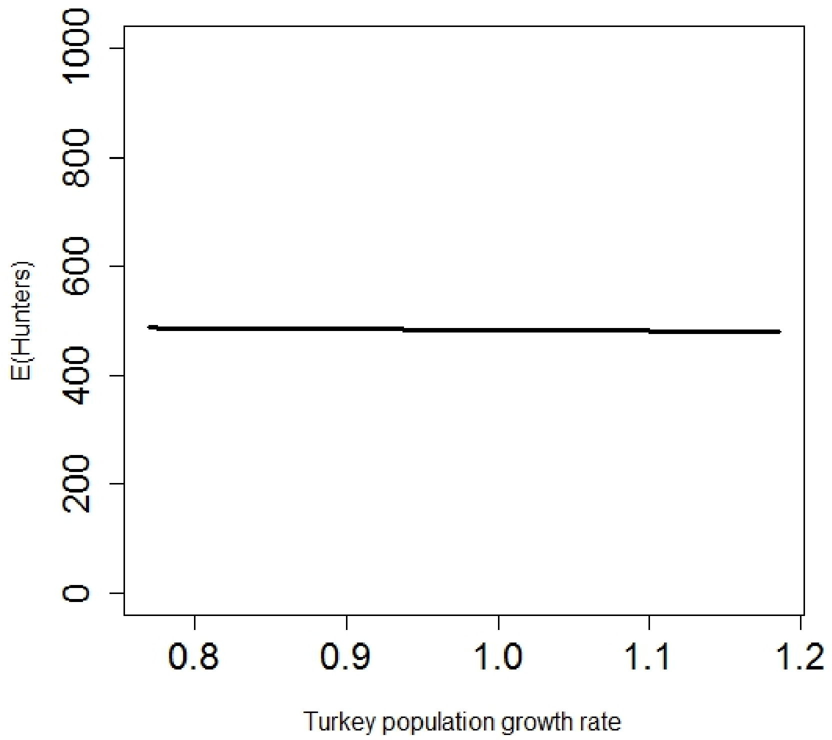

Expected number of hunters (E(Hunters)) was modeled as a function of the county-scale covariates human population size (A), proportion of successful hunters during the current years' spring hunting season (B), and the area of public lands open to hunting (C), as well as the management-unit scale covariates for the estimated density of male turkeys at the start of the current years' spring hunting season (D), and the estimated growth rate of the male turkey population at the start of spring hunting from 2 years prior to one year prior (E).
