# Supplementary material for: Spatial-temporal dynamics of hunter effort for wild turkeys in Michigan
Source: PLoS One. 2020 Apr 1;15(4):e0230747. doi: 10.1371/journal.pone.0230747 (PMC7112203; doi:10.1371/journal.pone.0230747)
Supplement: S1 Table — (PDF) [file pone.0230747.s005.pdf]

Table S1. Results of model-selection analyses to evaluate random-effects structures and statistical distributions for modeling drivers of estimated county-scale wild turkey hunter population size during spring and fall hunting seasons in southern Michigan, USA.

Season & model structure[a]	Δ AIC$_c$
Spring[b]	
Random intercepts (annual + county) and slopes (time trend): NB1	0
Random intercepts (county) and slopes (time trend): NB1	50.9
Random intercepts (annual + county) and slopes (time trend): NB2	76.0
Random intercepts (county) and slopes (time trend): NB2	117.9
Random intercepts (county): NB1	425.1
Random intercepts (county): NB2	690.5
Random intercepts (annual): NB1	1211.7
Random intercepts (annual): NB2	1376.3
Fall[c]	
Random intercepts (annual + county) and slopes (time trend): NB1	0
Random intercepts (county) and slopes (time trend): NB1	31.0
Random intercepts (county): NB1	265.4
Random intercepts (county) and slopes (time trend): NB2	326.8
Random intercepts (annual + county) and slopes (time trend): NB2	326.9
Random intercepts (annual): NB1	342.1
Random intercepts (county): NB2	425.0
Random intercepts (annual): NB2	480.9

We ranked and compared models using Akaike's Information Criterion corrected for small sample sizes (AIC$_C$). All subsequent fixed-effects model selection was conducted using the top random effects and distributional model shown here for each hunting season.

[a] Statistical distributions considered included the negative binomial model with a linear (NB1) and quadratic (NB2) variance-mean relationship [25,26]. In addition, random slopes indicated above for time trends were implemented at the county scale (i.e., varying time trends in estimated hunter population size by county).

[b] All spring models were fit using the global fixed effects model with the following covariates: quadratic time trends (i.e., Time + Time^2), season length, hunter success the previous spring, hunter success the previous fall, hunter interference, hunter satisfaction, area of public hunting land, human population size, turkey density, and turkey population growth.

[c] All spring models were fit using the global fixed effects model with the following covariates: quadratic time trends (i.e., Time + Time^2), hunter success the current year spring season, hunter success the previous fall, spring hunter interference, spring hunter satisfaction, area of public hunting land, human population size, turkey density, and turkey population growth.
