# Supplementary material for: Spatial-temporal dynamics of hunter effort for wild turkeys in Michigan
Source: PLoS One. 2020 Apr 1;15(4):e0230747. doi: 10.1371/journal.pone.0230747 (PMC7112203; doi:10.1371/journal.pone.0230747)
Supplement: S2 Table — (PDF) [file pone.0230747.s006.pdf]

Table S2. Results of preliminary assessment of effects of hunter perception and turkey population dynamics covariates on estimated county-scale spring turkey hunter population size in southern Michigan, USA (2001-2014).

Models[a]	Δ AIC_c
Hunter perception	
Hunter interference	0
Hunter success the previous fall	2
Hunter satisfaction	3
Hunter success the previous spring	3.2
Turkey population	
Population density	0
Population growth	5.1

We ranked and compared models using Akaike's Information Criterion corrected for small sample sizes (AIC_C). Final candidate fixed-effects model sets were generated using covariates ≤ of the top model for each group.

[a] All models used the top random-effects structure (Table S1) and thus included quadratic and county-specific time trends, and annual random intercepts.
