# Supplementary material for: Spatial-temporal dynamics of hunter effort for wild turkeys in Michigan
Source: PLoS One. 2020 Apr 1;15(4):e0230747. doi: 10.1371/journal.pone.0230747 (PMC7112203; doi:10.1371/journal.pone.0230747)
Supplement: S3 Table — (PDF) [file pone.0230747.s007.pdf]

Table S3. Results of preliminary assessment of effects of hunter perception and turkey population dynamics covariates on estimated county-scale fall turkey hunter population size in southern Michigan, USA (2002-2013).

Models[a]	Δ AIC_c
Hunter perception	
Hunter success the current spring	0
Hunter interference	2.6
Hunter success the previous fall	2.8
Hunter satisfaction	2.8
Turkey population	
Population density	0
Population growth	0.8

We ranked and compared models using Akaike's Information Criterion corrected for small sample sizes (AIC_C). Final candidate fixed-effects model sets were generated using covariates ≤ 2 AIC_C units of the top model for each group.

[a] All models used the top random-effects structure identified above (Table S1) and thus included quadratic and county-specific time trends, and annual random intercepts.
