# Supplementary material for: Spatial-temporal dynamics of hunter effort for wild turkeys in Michigan
Source: PLoS One. 2020 Apr 1;15(4):e0230747. doi: 10.1371/journal.pone.0230747 (PMC7112203; doi:10.1371/journal.pone.0230747)
Supplement: S4 Table — (PDF) [file pone.0230747.s008.pdf]

Table S4. Estimated county-scale annual growth rates (natural log scale) for spring and fall hunting seasons for turkey hunter populations in southern Michigan, USA.

	Spring			Fall		
County	Growth rate	Start	End	Growth rate	Start	End
Allegan	-0.006	2762	2450	-0.030	735	539
Barry	-0.032	2752	1727	-0.030	904	503
Bay	0.045	309	469	0.153	14	113
Berrien	0.061	453	901	-0.030	264	333
Branch	0.020	607	845	-0.030	286	225
Calhoun	-0.009	1490	1474	-0.030	814	452
Cass	-0.010	982	732	-0.030	302	311
Clinton	-0.017	1470	1176	-0.022	266	229
Eaton	-0.015	1105	933	-0.022	243	395
Genessee	0.088	470	1479	0.174	68	480
Gratiot	-0.009	1317	1048	-0.022	235	302
Hillsdale	0.030	1010	1460	0.108	128	321
Huron	0.013	1253	1476	0.653	0	466
Ingham	0.029	765	1429	0.108	137	437
Ionia	-0.022	1804	1454	-0.022	322	238
Isabella	-0.014	1670	1304	-0.025	403	330
Jackson	0.013	1583	2110	0.108	132	589
Kalamazoo	0.010	858	1225	-0.030	358	410
Kent	-0.009	2574	2303	0.073	380	583
Lapeer	0.011	1394	1783	0.174	200	744
Lenawee	0.058	532	1041	0.108	0	446
Livingston	0.056	585	1511	0.108	0	489
Macomb	0.160	90	586	0.174	0	243
Midland	-0.027	1524	1077	-0.025	499	381
Monroe[a]	0.165	75	607	NA	NA	NA
Montcalm	-0.030	2888	1943	-0.022	1070	360
Muskegon	-0.038	1690	777	0.073	254	254
Oakland	0.096	347	1124	0.174	64	385
Ottawa	0.052	856	1689	0.073	170	422
Saginaw	0.002	1814	1774	0.153	95	579
Sanilac	0.015	1220	1992	0.653	0	664
Shiawassee	0.022	957	1136	0.108	109	333
St. Clair	0.062	795	1653	0.174	0	601
St. Joseph	0.019	702	840	-0.030	269	225
Tuscola	-0.005	1978	2002	0.653	0	624
Van Buren	0.001	1199	1208	-0.030	679	354
Washtenaw	0.082	619	1665	0.108	0	465
Wayne[a]	0.645	0	146	NA	NA	NA

Total number of hunters estimated by county on the first (Start: spring = 2001, fall = 2002) and final (End: spring = 2014, fall = 2013) years of study are also provided.

[a] No fall hunting.
