# Supplementary material for: Spatial-temporal dynamics of hunter effort for wild turkeys in Michigan
Source: PLoS One. 2020 Apr 1;15(4):e0230747. doi: 10.1371/journal.pone.0230747 (PMC7112203; doi:10.1371/journal.pone.0230747)
Supplement: S5 Table — (PDF) [file pone.0230747.s009.pdf]

Table S5. Results of model selection analyses to evaluate support for hypothesized drivers of county-scale wild turkey hunter population size during spring and fall hunting seasons in southern Michigan, USA.

Season & model structure	Δ AIC_c	w_i
Spring[a]		
Trend + $Trend^2$ + Hunter Interference + Human Population + Turkey Density	0	0.14
Trend + $Trend^2$ + Hunter Success + Hunter Interference + Human Population + Turkey Density	0.4	0.12
Trend + $Trend^2$ + Human Population + Turkey Density	0.7	0.10
Trend + $Trend^2$ + Hunter Success + Human Population + Turkey Density	1	0.09
Trend + $Trend^2$ + Hunter Interference + Area of Public Land + Human Population + Turkey Density	1.8	0.06
Trend + $Trend^2$ + Season length + Hunter Interference + Human Population + Turkey Density	1.9	0.05
Trend + $Trend^2$ + Hunter Success + Hunter Interference + Area of Public Land + Human Population + Turkey Density	2.2	0.05
Trend + $Trend^2$ + Season length + Hunter Success + Hunter Interference + Human Population + Turkey Density	2.3	0.04
Trend + $Trend^2$ + Area of Public Land + Human Population + Turkey Density	2.5	0.04
Trend + $Trend^2$ + Season length + Human Population + Turkey Density	2.7	0.04
Fall[b]		
Trend + $Trend^2$ + Hunter Success + Human Population	0	0.09
Trend + $Trend^2$ + Hunter Success + Area of Public Land + Human Population	0	0.09
Trend + $Trend^2$ + Human Population	0.9	0.06
Trend + $Trend^2$ + Area of Public Land + Human Population	0.9	0.06
Trend + $Trend^2$ + Hunter Success	1	0.6
Trend + $Trend^2$ + Hunter Success + Area of Public Land	1	0.06
Trend + $Trend^2$ + Hunter Success + Area of Public Land + Human Population + Turkey Density	1.8	0.04
Trend + $Trend^2$ + Hunter Success + Human Population + Turkey Density	1.8	0.04
Trend + $Trend^2$ + Area of Public Land	1.8	0.04
Trend + $Trend^2$	1.8	0.04
Trend + $Trend^2$ + Hunter Success + Human Population + Lambda	1.9	0.04
Trend + $Trend^2$ + Hunter Success + Area of Public Land + Human Population + Lambda	2	0.03

We ranked and compared models using Akaike's Information Criterion corrected for small sample sizes (AIC_C) and normalized Akaike model weights (w_i).

[a] County-scale covariates represent a quadratic time trend across the study duration (Time + $Time^2$), proportion of successful turkey hunters during the previous fall hunting season (Hunter Success), proportion of turkey hunters during previous spring season that were not interfered with (Hunter Interference), human population size (Human Population), and the amount of public lands open to hunting (Area of Public Land). Covariates measured at the management-unit scale included length of the spring hunting season in days (Season Length) and estimated density of male turkeys at the start of the hunting season during the previous spring (Turkey Density).

[b] County-scale covariates represent a quadratic time trend across the study duration (Time + $Time^2$), proportion of successful turkey hunters during the current years' spring hunting season (Hunter Success), human population size (Human Population), and the amount of public lands open to hunting (Area of Public Land). Covariates measured at the management-unit scale included the estimated density of male turkeys at the start of the current years' spring hunting season (Turkey Density), and the estimated finite rate of change for male turkey population at the start of spring hunting from 2 years prior to one year prior (Lambda).
